# Computational pathology in 2030: a Delphi study forecasting the role of AI in pathology within the next decade

**DOI:** 10.1016/j.ebiom.2022.104427

**Published:** 2023-01-04

**Authors:** M. Alvaro Berbís, David S. McClintock, Andrey Bychkov, Jeroen Van der Laak, Liron Pantanowitz, Jochen K. Lennerz, Jerome Y. Cheng, Brett Delahunt, Lars Egevad, Catarina Eloy, Alton B. Farris, Filippo Fraggetta, Raimundo García del Moral, Douglas J. Hartman, Markus D. Herrmann, Eva Hollemans, Kenneth A. Iczkowski, Aly Karsan, Mark Kriegsmann, Mohamed E. Salama, John H. Sinard, J. Mark Tuthill, Bethany Williams, César Casado-Sánchez, Víctor Sánchez-Turrión, Antonio Luna, José Aneiros-Fernández, Jeanne Shen

**Affiliations:** a Department of R&D, HT Médica, San Juan de Dios Hospital, Córdoba, Spain; b Faculty of Medicine, Autonomous University of Madrid, Madrid, Spain; c Department of Laboratory Medicine and Pathology, Mayo Clinic, Rochester, MN, USA; d Department of Pathology, Kameda Medical Center, Kamogawa, Chiba, Japan; e Department of Pathology, Radboud University Medical Center, Nijmegen, the Netherlands; f Department of Pathology, University of Michigan, Ann Arbor, MI, USA; g Department of Pathology, Center for Integrated Diagnostics, Massachusetts General Hospital/Harvard Medical School, Boston, MA, USA; h Wellington School of Medicine and Health Sciences, University of Otago, Wellington, New Zealand; i Department of Oncology-Pathology, Karolinska Institute, Stockholm, Sweden; j Pathology Laboratory, Institute of Molecular Pathology and Immunology, University of Porto, Porto, Portugal; k Department of Pathology and Laboratory Medicine, Emory University, Atlanta, GA, USA; l Pathology Unit, Azienda Sanitaria Provinciale Catania, Gravina Hospital, Caltagirone, Italy; m Department of Pathology, San Cecilio Clinical University Hospital, Granada, Spain; n Department of Anatomic Pathology, University of Pittsburgh Medical Center, Pittsburgh, PA, USA; o Department of Pathology, Massachusetts General Hospital and Harvard Medical School, Boston, MA, USA; p Department of Pathology, Erasmus University Medical Center, Rotterdam, the Netherlands; q Department of Pathology, Medical College of Wisconsin, Milwaukee, WI, USA; r Department of Pathology & Laboratory Medicine, University of British Columbia, Michael Smith Genome Sciences Centre, Vancouver, Canada; s Institute of Pathology, University Hospital Heidelberg, Heidelberg, Germany; t Department of Pathology, Sonic Healthcare, Austin, TX, USA; u Department of Pathology, Yale University School of Medicine, New Haven, CT, USA; v Department of Pathology, Henry Ford Hospital, Detroit, MI, USA; w Department of Histopathology, Leeds Teaching Hospitals NHS Trust, Leeds, UK; x Department of Plastic and Reconstructive Surgery, La Paz University Hospital, Madrid, Spain; y Department of General Surgery and Digestive Tract, Puerta de Hierro-Majadahonda University Hospital, Madrid, Spain; z Department of Integrated Diagnostics, HT Médica, Clínica Las Nieves, Jaén, Spain; aa Department of Pathology and Center for Artificial Intelligence in Medicine & Imaging, Stanford University School of Medicine, Stanford, CA, USA

**Keywords:** Artificial intelligence, Machine learning, Digital pathology, Computational pathology, Anatomic pathology, Pathologist workflow

## Abstract

**Background:**

Artificial intelligence (AI) is rapidly fuelling a fundamental transformation in the practice of pathology. However, clinical integration remains challenging, with no AI algorithms to date in routine adoption within typical anatomic pathology (AP) laboratories. This survey gathered current expert perspectives and expectations regarding the role of AI in AP from those with first-hand computational pathology and AI experience.

**Methods:**

Perspectives were solicited using the Delphi method from 24 subject matter experts between December 2020 and February 2021 regarding the anticipated role of AI in pathology by the year 2030. The study consisted of three consecutive rounds: 1) an open-ended, free response questionnaire generating a list of survey items; 2) a Likert-scale survey scored by experts and analysed for consensus; and 3) a repeat survey of items not reaching consensus to obtain further expert consensus.

**Findings:**

Consensus opinions were reached on 141 of 180 survey items (78.3%). Experts agreed that AI would be routinely and impactfully used within AP laboratory and pathologist clinical workflows by 2030. High consensus was reached on 100 items across nine categories encompassing the impact of AI on (1) pathology key performance indicators (KPIs) and (2) the pathology workforce and specific tasks performed by (3) pathologists and (4) AP lab technicians, as well as (5) specific AI applications and their likelihood of routine use by 2030, (6) AI's role in integrated diagnostics, (7) pathology tasks likely to be fully automated using AI, and (8) regulatory/legal and (9) ethical aspects of AI integration in pathology.

**Interpretation:**

This systematic consensus study details the expected short-to-mid-term impact of AI on pathology practice. These findings provide timely and relevant information regarding future care delivery in pathology and raise key practical, ethical, and legal challenges that must be addressed prior to AI's successful clinical implementation.

**Funding:**

No specific funding was provided for this study.

**Research in context**

**Evidence before this study**

Although the list of publications in computational pathology/pathology AI (CPath/AI) continues to grow, very few algorithms are currently in routine clinical use in pathology, and there appears to be a significant "translation gap." To understand perceptions surrounding the role of AI in pathology and identify potential contributors to this translation gap, we searched PubMed for peer-reviewed journal and conference articles published between database inception and August 15, 2022, using the terms (“artificial intelligence” OR “machine learning” OR "deep learning" OR "computational pathology" OR "digital pathology") AND (“histo∗” OR “pathology") AND ("survey") across all fields. This yielded 351 results, of which 9 contained surveys related to CPath/AI. The studies we identified focused primarily on soliciting general opinions and attitudes regarding the integration of AI in pathology, whether participants were using CPath/AI algorithms and which algorithms they were using, or on the perceived promise of specific applications. We did not identify any comprehensive, systematic surveys providing detailed perspectives and insights regarding the full range of topics pertinent to the clinical application of AI in pathology, including technical, legal, regulatory, and ethical aspects. Furthermore, all surveys drew from heterogeneous participant pools comprising a wide range of backgrounds and experience levels, including many participants with non-medical or non-pathology backgrounds, and/or limited to no experience with CPath/AI. No surveys focused on eliciting the perspectives of clinically-active pathologists with dedicated expertise in CPath/AI, who might be best positioned to provide insights into the expected role of AI in pathology, including the most significant challenges that will need to be addressed in order to promote routine adoption.

**Added value of this study**

To address these gaps, we conducted a comprehensive Delphi consensus survey of international experts with specific experience developing and evaluating CPath/AI algorithms, almost all of whom are pathologists in active clinical practice. The goals of the survey were to: (1) investigate the expected impact of AI on pathology; (2) forecast the extent of clinical AI implementation in the specialty within the next decade; and (3) provide specific insights into which technical, legal, regulatory, and ethical aspects of AI integration would require the most attention in the coming years. Our survey encompassed nine topical categories, including the expected impact of AI on (1) pathology key performance indicators, (2) the pathology workforce, (3) specific tasks performed by pathologists, and (4) specific tasks performed by pathology laboratory technicians, as well as (5) specific AI applications and their likelihood of routine adoption by 2030, (6) the role of AI in integrated diagnostics, (7) pathology tasks which were likely to be fully automated using AI, and finally, (8) regulatory/legal and (9) ethical aspects of AI integration in pathology.

**Implications of all the available evidence**

There is strong consensus that AI will have a significant impact on the specialty of pathology within the coming decade, particularly with regard to improved diagnostic accuracy. Several algorithms are expected to be in routine use by the year 2030, including some that will fully replace pathologists on specific tasks. However, a lack of consensus remains regarding the anticipated impact of AI on diagnostic time and cost efficiency, pathologist diagnostic behaviour, and patient satisfaction, as well as many regulatory, legal, and ethical aspects related to AI integration. Our results highlight the need for prospective clinical trials examining the impact of CPath/AI algorithms on these key performance indicators, as well as the critical importance of addressing current regulatory, legal, and ethical barriers to the responsible adoption of AI in pathology in the coming decade.

## Introduction

Artificial intelligence (AI) is set to fuel an unprecedented transformation in healthcare by contributing to more accurate diagnoses, more agile, cost-effective, and standardized clinical workflows, and more effective and personalized treatments.^1^^,^^2^ Excitement and expectations regarding its potential have continued to build, as evidenced by the growing list of medical AI publications (from just 203 articles in 2005 to 12,563 in 2019).^2^, ^3^, ^4^, ^5^, ^6^, ^7^, ^8^ Pathology has attracted attention as an image-rich specialty likely to be strongly impacted by advances in AI and was recently the most-published specialty among 17 specialties engaged in medical AI research.^2^ The development of machine learning-based tools for image analysis has led to a surge in AI applications promising to revolutionize pathology workflows, and the advent of a new field, computational pathology (CPath).^4^ Key examples of AI application in anatomic pathology (AP) include automated assessment of prognostic biomarkers such as Ki-67 in breast cancer,^9^ tumour grading in prostate cancer,^10^^,^^11^ diagnosis of metastatic breast cancer in lymph nodes,^12^ and optimization of clinical laboratory workflows, such as automated quality control (QC).^13^^,^^14^

However, few algorithms are currently in routine clinical use,^15^ and there is a dearth of studies evaluating their impact in clinical settings.^16^ Simultaneously, ethical concerns have been raised regarding potential data privacy breaches, systemic algorithmic bias, harm related to erroneous AI-generated outputs, and exacerbation of healthcare disparities.^17^ Along with hurdles related to regulatory approval and reimbursement for AI products, these have contributed to a significant AI "translation gap" in pathology,^15^ which we define as the failure to prospectively validate and successfully integrate AI models into real-world clinical workflows. Although various opportunities and challenges surrounding AI in pathology have been extensively discussed in the literature, to date, there has been no systematic survey regarding this topic from the short-to-medium term perspective of digital and CPath experts. To address this gap, we conducted a consensus survey to gain detailed insight into the current challenges, expectations, and perspectives surrounding the role of AI in pathology, from the standpoint of an international panel of "early adopters", most of them pathologists in active clinical practice with first-hand experience developing and evaluating the clinical utility of AI algorithms. For this survey, we applied the Delphi method, a robust, widely accepted tool for building consensus among experts^18^ which has outperformed standard statistical methods.^19^

Our goals were to: 1) investigate the expected impact of AI on pathology; 2) forecast the extent of clinical AI implementation by 2030; and 3) provide specific insights into which technical, legal, regulatory, and ethical aspects of AI integration will require the most attention in the coming years. We expect the results of this study to be of broad interest to a wide range of digital health professionals.

## Methods

### Expert panel recruitment

**Fig. 1:**
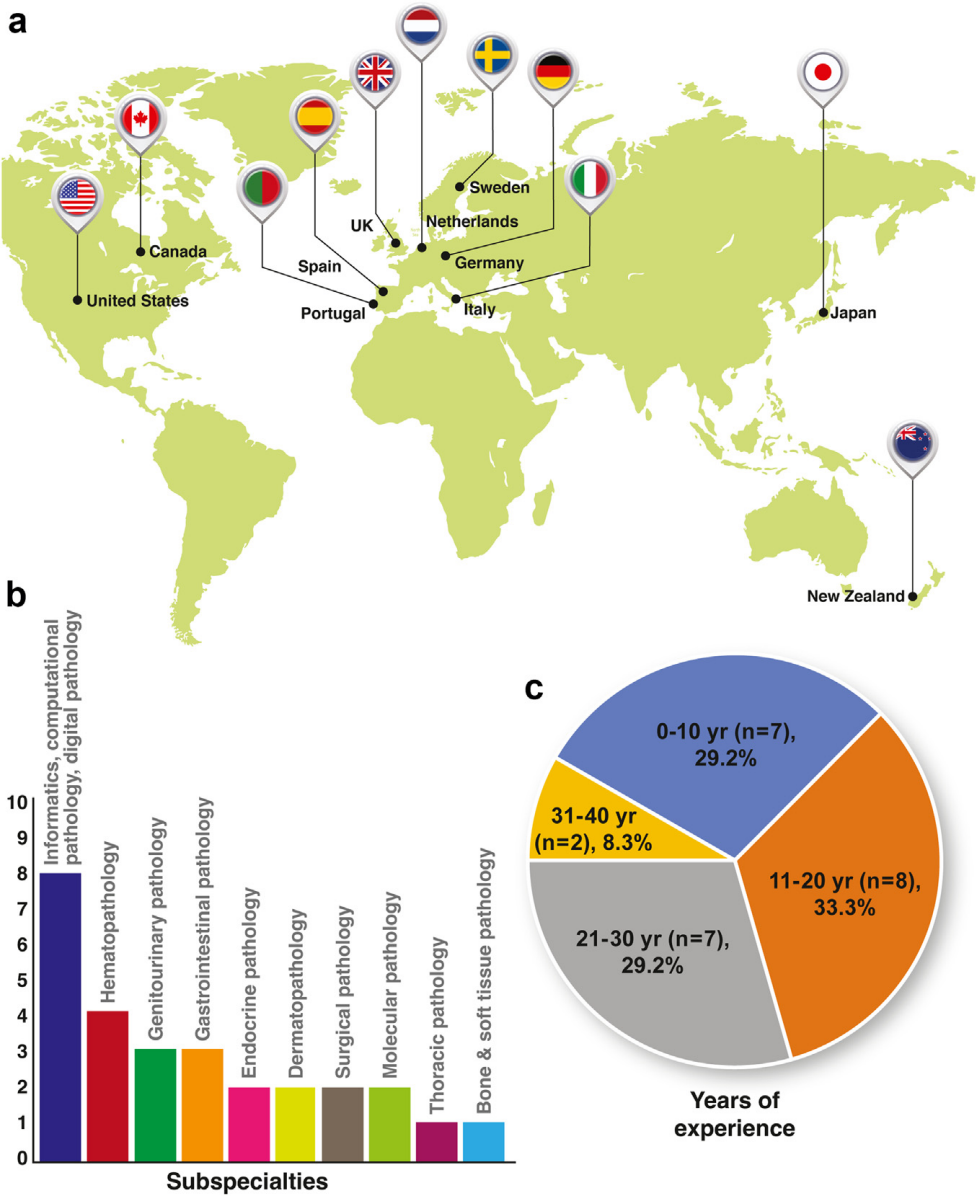
**Characteristics of the expert panel (n** = **24)**. (a) The map shows the countries represented. (b) The bar chart shows the area(s) of subspecialisation represented, with the number of panellists within each subspecialty shown on the y-axis (multiple responses possible per panellist). (c) The pie chart shows the number (n) and percentage (%) of panellists falling within each practice experience subgroup (0–10 years, 11–20 years, 21–30 years, and 31–40 years of practice experience, respectively).

The panelist recruitment criteria were: 1) pathology (anatomic and/or laboratory medicine) professionals with an MD (or equivalent medical degree) and/or PhD, and 2) authorship of at least one PubMed-indexed CPath/AI publication between 2016 and 2020. Two members of the research team (M.A.B. and J.A-F.) identified prospective candidates through the research team's professional network and by reviewing the websites of relevant professional organizations (Digital Pathology Association, Association of Pathology Informatics, European Society for Digital and Integrative Pathology, etc.) followed by confirmation of the inclusion criteria. We aimed to recruit a minimum of 15 panellists for the study. Assuming a 50% participation rate, we estimated that at least 30 invitations needed to be made. Since the general agreement is that the larger the panel size, the more reliable the group judgments, we therefore opted to invite all potential candidates (39 in total) who met the inclusion criteria for the study. One research team member (J.A-F.) sent the same invitation email (Fig. S1) to the 39 candidates in December 2020, of whom nine were women and 30 were men. By country of residence, 22 were practicing in the USA, 12 in Europe, two in Canada, two in Japan, and one in New Zealand. Failure to respond to a second invitation email was interpreted as declining participation in the survey. A total of 24 experts (62%) accepted and completed all three rounds (100%) of the survey, of whom 4 (16.7%) were female (additional panellist characteristics summarized in Fig. 1 and Table S1).

### Delphi study procedure

**Fig. 2:**
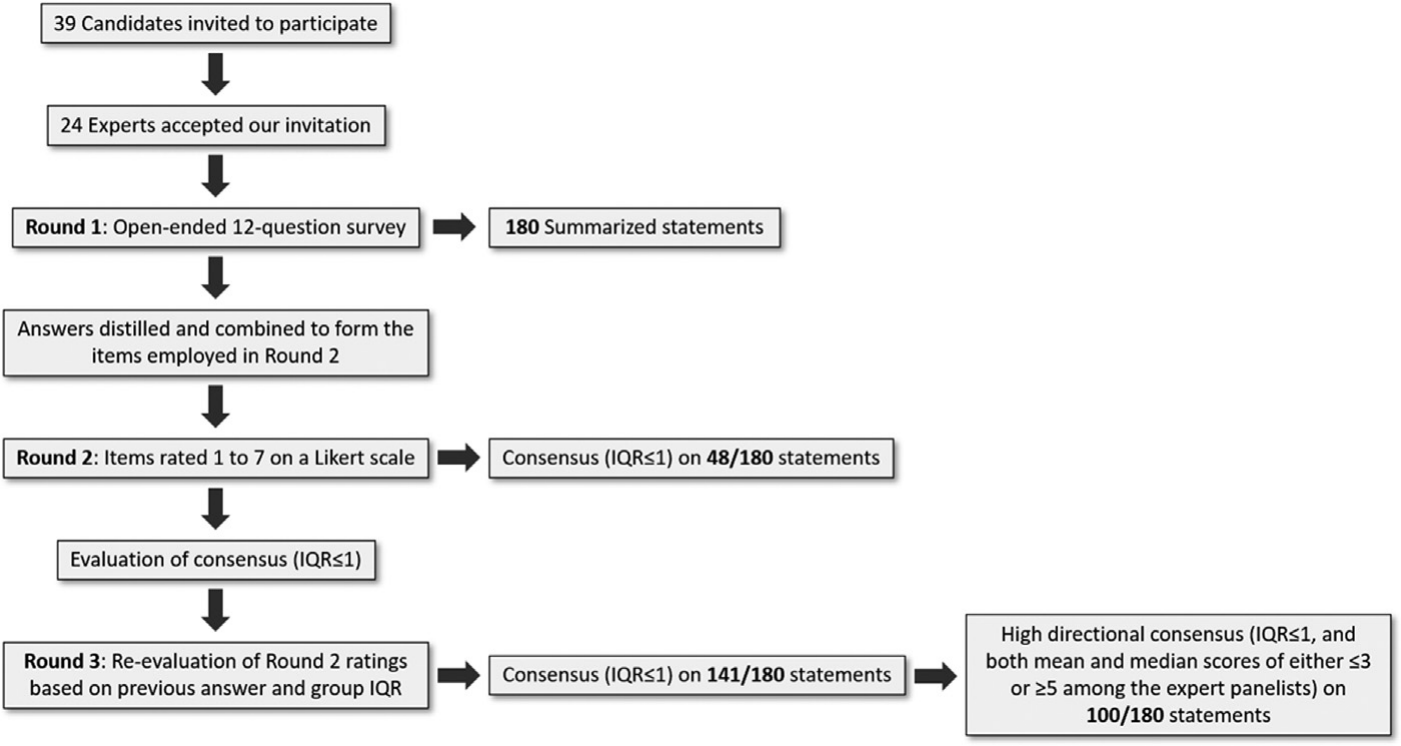
**Flowchart illustrating the Delphi study process**.

This Delphi study was conducted over three rounds (Fig. 2) via a series of questionnaires combined with controlled opinion feedback.^20^^,^^21^ In Round 1, a preliminary review of the pathology AI literature was performed as follows: a member of the research team (M.A.B.) searched the Pubmed database using the search terms [(computational AND pathology) OR (pathology AND artificial intelligence) OR (artificial intelligence AND whole slide image) OR (deep learning AND pathology) OR (deep learning AND whole slide image)] in all fields for publications from the last five years (between August 15, 2015 and August 15, 2020), yielding approximately 78,000 results, which were then triaged to identify publications relevant to CPath/AI based on title and abstract content. Two members of the research team (M.A.B. and J.A-F.) then jointly reviewed the abstracts or full-text (as necessary) to extract relevant topics which formed the basis of the open-ended questions in Round 1. In combination with the research team's empirical experience, these were used to generate an open-ended questionnaire containing 12 questions regarding the following topics: 1) forecasting the future of AI in pathology, 2) specific pathology AI applications, and 3) ethical and regulatory aspects (Table S2). The response period for the Round 1 questionnaire was December 8–21, 2020. Following completion of the Round 1 questionnaire by all panellists, two research team members (M.A.B. and J.A-F.) either directly reproduced or combined and distilled the panellist responses into the statements comprising the questionnaire items used in subsequent rounds.

In Round 2, the panellists rated each item on a 7-point Likert scale, with different scores designed to fit different question categories (i.e., a score of 1 indicating “Impossible” regarding event likelihood, and a score of 7 indicating “Certain”) with higher scores generally representing more favourable opinions toward the future role or impact of AI on Pathology (Table S3). Responses for Round 2 were collected from January 12–30, 2021.

In Round 3, the panellists were asked to re-rate all items not reaching consensus (defined as an interquartile range (IQR)≤1 for ratings along the Likert scale^21^) during Round 2. They were shown their Round 2 ratings on each item with the group median and IQR, and given the option to change their previous ratings, if desired. Responses for Round 3 were collected from February 21-March 24, 2021.

All questionnaires were completed via a Google Forms (Google Inc, USA) survey sent to each participant via an individualized link. The response data were automatically stored in a linked response spreadsheet in Google Sheets (Google Inc, USA), which was then exported in .csv format for downstream data analysis. Participants remained anonymous to one another during all three Rounds, with each participant able to view only their own responses during Rounds 1 and 2, and the anonymized group medians and IQRs during Round 3.

### Ethics statement

Formal institutional review board/ethical committee approval was not required for this study, as it did not involve any patient data collection or impact on patient care. Written (email) agreement by the expert panellists to participate in the survey was accepted as informed consent. All survey rounds were completed by the panellists anonymously. Participation was entirely voluntary, and there was no financial compensation for study participation and no disadvantage related to non-participation.

### Statistical analysis

Wilcoxon rank-sum exact tests (two-tailed, alpha = 0.05) were performed using STATA v16 (StataCorp LLC, USA) to examine for significant differences in panellist scores by practice location, pathology subspecialty, and years in practice.

### Role of funders

Not applicable; no specific funding was provided for this study.

## Results

### Survey rounds

The unstructured Round 1 allowed the panellists freedom in expressing their thoughts on topics they felt were relevant to AI in pathology over the next decade. This generated 180 summative statements spanning nine domains: (1) key performance indicators (KPIs), (2) the pathology workforce, (3) pathologist tasks, (4) technician tasks, (5) specific AI applications, (6) role of AI in integrated diagnostics, (7) tasks likely to be fully automated by AI, and (8) regulatory/legal and (9) ethical aspects of AI integration (Table S4).

The Delphi method allows the achievement of greater consensus (a reduction in variance across rounds, as measured by the IQR) among a limited number of experts. All 24 participants completed all 180 survey questions. There were no missing data, and no data cleaning was performed prior to analysis. After Round 2, responses to 48 (26.7%) statements reached consensus (IQR≤1), further increasing to 141 (78.3%) after Round 3. Mean and median Likert scores for each statement ranged between 3.04-6.83 and 3–7, respectively. Table 1, Table 2, Table 3, Table 4, Table 5, Table 6 present the 100 statements which achieved high directional consensus (defined as IQR≤1, *and* both mean and median scores of either ≤3 or ≥5). For these, two-tailed Wilcoxon rank sum tests demonstrated no significant differences in Likert scores between the comparison groups (North American vs. non-North American, Informatics or Digital/CPath vs. other subspecialty, and ≤10 years vs. ≥11 years in practice) on 85 statements. The remaining statements are further discussed in the sections below, which summarize the most significant survey results across the nine domains (Table S4).

**Table 1:** Statements on which high directional consensus was reached regarding the impact of AI on pathology key performance indicators (KPIs).

By 2030, due to the integration of AI in the pathology setting …					
Key performance indicator	Item #	Mode (%)	Mean (SD)	Median (IQR)	Result
Standardization of pre-analytical processes (staining and slicing techniques) will increase	3	5 (41.7)	5.38 (0.92)	5.0 (5.0–6.0)	Agree
Diagnostic accuracy will increase	6	6 (58.3)	5.67 (1.05)	6.0 (5.0–6.0)	Strongly agree
Diagnosis and grading of tumors will be more standardized, bringing more objectivity to the diagnosis of certain entities that are currently subject to high interobserver variability	7	6 (62.5)	6.04 (0.62)	6.0 (6.0–6.0)	Strongly agree
Detection of rare events (small metastases, small tumor foci) will increase	8	6 (62.5)	5.88 (1.03)	6.0 (6.0–6.0)	Strongly agree
Analyses will be more quantitative	9	6 (45.8)	6.21 (0.72)	6.0 (6.0–7.0)	Strongly agree
Completeness of reports will increase	10	5 (54.2)	5.13 (1.03)	5.0 (5.0–6.0)	Agree
Complexity of reports will increase	11	5 (50.0)	5.13 (1.12)	5.0 (5.0–6.0)	Agree
Quality of reports will increase	12	5 (33.3)	5.38 (1.24)	5.0 (5.0–6.0)	Agree

For the Mode, (%) designates the percentage of panellists who selected that score. AI, artificial intelligence; SD, standard deviation; IQR, interquartile range. Item # refers to the question # on the survey questionnaire. Likert scale interpretation: *Agreement level*: 1 = Very strongly disagree, 2 = Strongly disagree, 3 = Disagree, 4 = Neither agree nor disagree, 5 = Agree, 6 = Strongly agree, 7 = Very strongly agree.

**Table 2:** Statements on which high directional consensus was reached regarding the impact of AI on the pathology workforce and associated tasks.

Task	Item #	Mode (%)	Mean (SD)	Median (IQR)	Involvement/agreement level
**By 2030, due to the integration of AI in the pathology setting …**					
The number of jobs for IT staff will …	18	5 (50.0)	5.54 (0.93)	5.0 (5.0–6.0)	Somewhat increase
The number of specialized “computational” pathologists will …	22	6 (45.8)	5.75 (0.79)	6.0 (5.0–6.0)	Greatly increase
Pathologists will be more involved in diagnostic tumor boards	44	6 (54.2)	5.58 (1.06)	6.0 (5.0–6.0)	Strongly agree
Pathologists will be more involved in multidisciplinary conferences	45	6 (58.3)	5.63 (1.06)	6.0 (5.0–6.0)	Strongly agree
Pathologists will be more involved in research activities	46	5 (37.5)	5.42 (1.06)	5.0 (5.0–6.0)	Agree
Pathologists will be spending more time in the study of rare lesions	47	5 (45.8)	5.13 (1.03)	5.0 (5.0–6.0)	Agree
**By 2030, the degree of involvement of pathologists in these tasks will be …**					
Digital pathologic diagnosis without the use of physical glass slides	29	6 (50.0)	5.58 (1.64)	6.0 (5.5–6.5)	Routine
Interpretation of computationally derived measurements and evaluations	30	6 (45.8)	6.08 (1.10)	6.0 (6.0–7.0)	Routine
Collaboration with EHR teams regarding the use of laboratory data for a wide range of clinical decision support tools	31	6 (45.8)	5.25 (1.03)	5.5 (5.0–6.0)	Routine
Evaluating different kinds of AI software and deciding whether these are appropriate for their workflow	35	6 (62.5)	5.54 (1.14)	6.0 (5.0–6.0)	Routine
Validation and QA/QC of AI solutions	36	6 (58.3)	5.63 (1.13)	6.0 (5.0–6.0)	Routine
Validation and QA/QC of AI-rendered diagnoses	37	6 (50.0)	5.88 (1.23)	6.0 (6.0–7.0)	Routine
Defining new categories of patients, based on new data made available through AI	38	5 (41.7)	5.04 (1.43)	5.0 (5.0–6.0)	Often
**By 2030, the degree of involvement of pathology laboratory technicians in these tasks will be …**					
Operation of digital slide scanners, digitization, and image management	48	7 (58.3)	6.25 (1.22)	7.0 (6.0–7.0)	Daily
QA/QC of digitized images	49	7 (50.0)	6.08 (1.41)	6.5 (6.0–7.0)	Daily
Digital pathology support for pathologists and other users, such as device calibration	50	6 (54.2)	5.88 (1.12)	6.0 (6.0–6.5)	Routine
Assessing histology consistency, i.e., re-addressing SOPs to make slides and corresponding images more suitable for AI (more consistent tissue and staining quality)	51	6 (62.5)	5.83 (0.70)	6.0 (5.5–6.0)	Routine
Validation and QA/QC of AI-rendered diagnoses	56	5 (45.8)	5.17 (0.96)	5.0 (5.0–6.0)	Often

For the Mode, (%) designates the percentage of panellists who selected that score. AI, artificial intelligence; SD, standard deviation; IQR, interquartile range; IT, information technology; EHR, electronic health record; QA/QC, quality assurance/quality control; SOP, standard operating procedure. Item # refers to the question # on the survey questionnaire. Likert scale interpretations: *Job number variation*: 1 = Dramatically decrease, 2 = Greatly decrease, 3 = Somewhat decrease, 4 = Remain the same, 5 = Somewhat increase, 6 = Greatly increase, 7 = Dramatically increase; *Agreement level*: 1 = Very strongly disagree, 2 = Strongly disagree, 3 = Disagree, 4 = Neither agree nor disagree, 5 = Agree, 6 = Strongly agree, 7 = Very strongly agree; *Involvement level*: 1 = Not involved at all, 2 = Rarely, 3 = Somewhat, 4 = Sometimes, 5 = Often, 6 = Routine, 7 = Daily.

**Table 3:** Statements on which high directional consensus was reached regarding AI applications in pathology.

By 2030, the probability of these AI tools being routinely used in pathology labs is …					
AI application	Item #	Mode (%)	Mean (SD)	Median (IQR)	Likelihood
Identification of micrometastases	78	7 (50.0)	6.17 (1.09)	6.5 (6.0–7.0)	Certain
Detection of lymph node metastases	79	7 (54.2)	6.33 (0.87	7.0 (6.0–7.0)	Certain
Quantification of IHC or IF stains, such as Ki-67, ER, PgR, PD-L1	85	7 (70.8)	6.67 (0.56)	7.0 (6.0–7.0)	Certain
Quantification of number of mitoses in H&E-stained images	86	7 (50.0)	6.33 (0.76)	6.5 (6.0–7.0)	Certain
Counting lymphocytes	87	7 (50.0)	6.42 (0.65)	6.5 (6.0–7.0)	Certain
Automated ordering of IHC for specific applications/assisting with selection of immunohistochemical stains needed	61	6 (45.8)	5.46 (0.93)	6.0 (5.0–6.0)	Very likely
Automated QA/QC of IHC positive and negative controls	62	6 (54.2)	5.75 (0.90)	6.0 (5.0–6.0)	Very likely
Proposing specific IHC or other molecular methods to solve a specific diagnostic problem	68	6 (41.7)	5.17 (1.34)	5.5 (5.0–6.0)	Very likely
Prioritization of cases (such as cases with neoplasia and infectious organisms in immunosuppressed patients)	69	6 (45.8)	5.50 (1.10)	6.0 (5.0–6.0)	Very likely
Quality control of whole-slide images (scanning process), and detection of poor-quality slides (tissue folds, poor staining)	73	6 (66.7)	6.13 (0.68)	6.0 (6.0–6.5)	Very likely
Quality improvement of whole-slide images	74	6 (62.5)	6.00 (0.92)	6.0 (6.0–6.5)	Very likely
Pre-selecting regions of interest suspicious for cancer for pathologists to view	76	7 (45.8)	6.29 (0.75)	6.0 (6.0–7.0)	Very likely
Identification of hotspot areas	77	7 (45.8)	6.25 (0.85)	6.0 (6.0–7.0)	Very likely
Detection of microorganisms (AFB, *H. pylori*)	81	6 (58.3)	6.17 (0.87)	6.0 (6.0–7.0)	Very likely
Assisting with tumor grading	82	6 (62.5)	6.21 (0.59)	6.0 (6.0–7.0)	Very likely
Quantification of eosinophils in eosinophilic esophagitis	88	6 (54.2)	6.13 (0.68)	6.0 (6.0–7.0)	Very likely
Quantitation of features (e.g., fibrosis in various organs, liver steatosis, etc.)	89	6 (62.5)	6.29 (0.55)	6.0 (6.0–7.0)	Very likely
Marking of perineural invasion, lymphovascular invasion	90	6 (50.0)	5.79 (0.98)	6.0 (5.0–6.0)	Very likely
Automated measurements (e.g., of tumor areas)	94	6 (54.2)	6.21 (0.66)	6.0 (6.0–7.0)	Very likely
Ensuring all diagnostically relevant areas on the slide are viewed prior to report finalization	95	6 (50.0)	5.42 (0.83)	6.0 (5.0–6.0)	Very likely
Mandatory 2nd reads when the pathologist diagnosis doesn't match the potential AI diagnosis (within a predefined range/%; e.g., if the AI tool detects potential tumor on a biopsy but the pathologist reads the biopsy as no evidence of tumor)	97	6 (54.2)	5.79 (0.83)	6.0 (5.0–6.0)	Very likely
Standardization of pathology reports	98	6 (66.7)	5.88 (0.68)	6.0 (6.0–6.0)	Very likely
AI-assisted laboratory workflow management, including workload assignments to pathologists, residents, and technicians	59	5 (45.8)	5.33 (1.31)	5.0 (5.0–6.0)	Likely
Detection of signet ring-cell cancer	80	5 (42.7)	5.29 (1.08)	5.0 (5.0–6.0)	Likely
Pre-selection of potentially cancer-positive samples for pathologist's review, while the bulk of clearly negative samples can be automatically processed	63	5 (45.8)	5.13 (1.26)	5.0 (5.0–6.0)	Likely
Triaging of cases to the most appropriate pathologist at the earliest possible time	64	5 (41.7)	5.08 (1.41)	5.0 (5.0–6.0)	Likely
Providing a set of differential diagnoses on difficult cases	92	5 (45.8)	5.13 (0.90)	5.0 (5.0–6.0)	Likely
Proposing specific additional tests for solving a diagnostic problem (e.g., AI algorithm suggesting STAT6 immunostaining on a spindle cell neoplasm of the pleura)	93	6 (37.5)	5.17 (1.24)	5.0 (5.0–6.0)	Likely
Import of contextually-related data on a case for quick review by the pathologist during diagnostic slide review	96	5 (58.3)	5.21 (0.72)	5.0 (5.0–6.0)	Likely
Pre-populating relevant report details from the medical record/gross description	99	5 (54.2)	5.29 (0.95)	5.0 (5.0–6.0)	Likely
Selection of the appropriate synoptic report based on prior pathology findings, including the current case gross report	100	5 (50.0)	5.38 (0.88)	5.0 (5.0–6.0)	Likely
Pre-populating reports based on AI interpretation of images	101	5 (45.8)	5.13 (1.03)	5.0 (5.0–6.0)	Likely
Finding the source of contaminants	102	5 (58.3)	5.17 (0.96)	5.0 (5.0–5.5)	Likely

For the Mode, (%) designates the percentage of panellists who selected that score. AI, artificial intelligence; SD, standard deviation; IQR, interquartile range; IHC, immunohistochemistry; IF, immunofluorescence; ER, oestrogen receptor; PgR, progesterone receptor; PD-L1, programmed cell death ligand 1; H&E, haematoxylin and eosin; QA/QC, quality assurance/quality control; AFB, acid-fast *Bacillus; H. pylori, Helicobacter pylori;* STAT6, signal transducer and activator of transcription 6. Item # refers to the question # on the survey questionnaire. Likert scale interpretation: *Likelihood*: 1 = Impossible, 2 = Very unlikely, 3 = Unlikely, 4 = Even chance/neutral, 5 = Likely, 6 = Very likely, 7 = Certain.

**Table 4:** Statements on which high directional consensus was reached regarding the role of AI in integrated diagnostics.

By 2030, the probability of these integrated diagnostic applications being used routinely is …					
AI application	Item #	Mode (%)	Mean (SD)	Median (IQR)	Likelihood
Identification of histologic regions to be sampled for genomic testing	104	5 (45.8)	5.38 (1.13)	5.0 (5.0–6.0)	Likely
Prediction of biomarker status and clinical outcomes for personalized medicine, based on integrated diagnostics	109	5 (58.3)	5.08 (1.14)	5.0 (5.0–5.5)	Likely
Selection of patients with prostate cancer for active surveillance versus radiotherapy/surgery, based on integration of pathology and radiology data	118	5 (54.2)	5.00 (1.22)	5.0 (5.0–6.0)	Likely
Creation of new categories of patients by integrating all “big data” from pathology, clinical lab, radiology, and genomics	119	5 (58.3)	5.04 (1.16)	5.0 (5.0–5.0)	Likely
Building risk stratification (prognostic) roadmaps for individual patients based on input from histology, radiology, and genomics	120	5 (54.2)	5.13 (0.99)	5.0 (5.0–6.0)	Likely
Use of integrated reports for select conditions, e.g., prostate cancer	121	5 (33.3)	5.33 (1.31)	5.0 (5.0–6.0)	Likely

For the Mode, (%) designates the percentage of panellists who selected that score. AI, artificial intelligence. SD, standard deviation; IQR, interquartile range. Item # refers to the question # on the survey questionnaire. Likert scale interpretation: *Likelihood*: 1 = Impossible, 2 = Very unlikely, 3 = Unlikely, 4 = Even chance/neutral, 5 = Likely, 6 = Very likely, 7 = Certain.

**Table 5:** Statements on which high directional consensus was reached regarding pathology tasks expected to be fully automated by 2030.

By 2030, the probability of these tasks being fully delegated to AI in pathology labs is …					
Task	Item #	Mode (%)	Mean (SD)	Median (IQR)	Likelihood
Verification of positive and negative controls for IHC	124	6 (58.3)	5.71 (0.91)	6.0 (5.0–6.0)	Very likely
Prioritization of cases	125	6 (50.0)	5.54 (1.47)	6.0 (5.0–6.0)	Very likely
Triage of cases to appropriate pathologists	126	6 (45.8)	5.46 (1.25)	6.0 (5.0–6.0)	Very likely
Contextual data lookup on patients from the EHR relevant to the pathology case being reviewed	127	6 (50.0)	5.25 (1.15)	6.0 (5.0–6.0)	Very likely
Slide QC (e.g., detection of tissue folds and tears, stain quality evaluation, etc.)	128	6 (58.3)	5.88 (1.03)	6.0 (6.0–6.0)	Very likely
Screening for microorganisms, such as AFB and *H. pylori*	129	6 (58.3)	5.96 (0.75)	6.0 (6.0–6.0)	Very likely
Screening of colorectal polyps	130	6 (41.7)	5.58 (1.02)	6.0 (5.0–6.0)	Very likely
Cervical cytology screening	131	7 (41.7)	6.21 (0.78)	6.0 (6.0–7.0)	Very likely
Screening lymph nodes for metastases	132	6 (54.2)	5.83 (0.76)	6.0 (5.0–6.0)	Very likely
Measurement tasks	135	6, 7 (41.7)	6.17 (0.92)	6.0 (6.0–7.0)	Very likely
Quantification of IHC or IF stains, such as Ki-67, ER, PgR, PD-L1	137	6 (45.8)	6.29 (0.69)	6.0 (6.0–7.0)	Very likely
Quantification of mitotic count on H&E-stained images	138	6 (50.0)	6.08 (0.72)	6.0 (6.0–7.0)	Very likely
Bone marrow differential counts	139	6 (37.5)	5.54 (1.02)	6.0 (5.0–6.0)	Very likely
MIB-1 scoring	141	6 (54.2)	6.04 (0.91)	6.0 (6.0–7.0)	Very likely
Assessing extent of liver steatosis and fibrosis	143	6 (41.7)	5.54 (1.14)	6.0 (5.0–6.0)	Very likely
Screening of tissues with a cancer diagnosis to select regions for tissue coring or macroscopic dissection	122	5 (58.3)	5.08 (1.02)	5.0 (5.0–5.5)	Likely
Slide screening for regions of interest	134	5 (50.0)	5.13 (0.99)	5.0 (5.0–6.0)	Likely
Grading of breast cancer	145	5 (33.3)	5.42 (1.14)	5.0 (5.0–6.0)	Likely
Grading of colorectal cancer	146	5 (37.5)	5.33 (1.09)	5.0 (5.0–6.0)	Likely

For the Mode, (%) designates the percentage of panellists who selected that score. AI, artificial intelligence; SD, standard deviation; IQR, interquartile range; IHC, immunohistochemistry; EHR, electronic health record; QC, quality control; AFB, acid-fast *Bacillus; H. pylori, Helicobacter pylori;* IF, immunofluorescence; ER, oestrogen receptor; PgR, progesterone receptor; PD-L1, programmed cell death ligand 1; H&E, haematoxylin and eosin. Item # refers to the question # on the survey questionnaire. Likert scale interpretation: *Likelihood*: 1 = Impossible, 2 = Very unlikely, 3 = Unlikely, 4 = Even chance/neutral, 5 = Likely, 6 = Very likely, 7 = Certain.

**Table 6:** Statements on which high directional consensus was reached regarding regulatory and ethical aspects.

By 2030, regarding the integration of AI in pathology …					
Aspect	Item #	Mode (%)	Mean (SD)	Median (IQR)	Likelihood
A set of new guidelines will be developed, specifically addressing the integration of AI in pathology	150	7 (79.2)	6.63 (0.82)	7.0 (7.0–7.0)	Very strongly agree
Specific validation procedures for different types of AI tools will be defined by regulatory bodies	151	7 (58.3)	6.46 (0.72)	7.0 (6.0–7.0)	Very strongly agree
The introduction of AI-based diagnostic modalities will require regulatory supervision, both related to the quality of the rendered diagnosis and the ultimate destination of the diagnostic information	161	7 (87.5)	6.83 (0.48)	7.0 (7.0–7.0)	Very strongly agree
As long as AI is used as a supportive method, ethical issues will be minor. However, when AI takes over tasks from the pathologist, i.e., making a diagnosis without human oversight, it will face major ethical challenges.	166	7 (75.0)	6.58 (0.93)	7.0 (6.5–7.0)	Very strongly agree
Pathologists will still be legally responsible for diagnoses made with the help of AI	173	7 (62.5)	6.25 (1.39)	7.0 (6.0–7.0)	Very strongly agree
Meeting regulatory requirements for most AI applications will be a lengthy and costly process, as it will involve large-scale prospective studies	157	5 (37.5)	5.46 (1.25)	5.5 (5.0–6.0)	Strongly agree
Definition of endpoints for clinical validation studies will be a common problem	158	6 (37.5)	5.50 (1.14)	6.0 (5.0–6.0)	Strongly agree
Post-marketing surveillance will pose important challenges, due to algorithm drift	159	6 (41.7)	5.50 (1.06)	6.0 (5.0–6.0)	Strongly agree
Regulatory approval of AI tools used for definitive (primary) diagnosis will be very strict, but AI used for advisory purposes (secondary) will also have to meet strict regulatory conditions	162	6 (70.8)	6.04 (0.55)	6.0 (6.0–6.0)	Strongly agree
CLIA regulations and clarification surrounding the use of laboratory data within pathology and laboratory processes versus outside of the laboratory will be reviewed and updated	163	6 (54.2)	5.63 (0.97)	6.0 (5.0–6.0)	Strongly agree
Governments will actively promote innovation in the areas of AI and medicine, fostering the advancement of AI in pathology	164	6 (58.3)	5.88 (0.74)	6.0 (5.0–6.0)	Strongly agree
Legal disputes will often arise regarding who should assume liability (pathologist, institution, developer, commercial vendor …) for diagnostic errors induced by AI	165	6 (41.7)	5.67 (1.05)	6.0 (5.0–6.0)	Strongly agree
AI and technology will be included in the educational curricula for medical students, pathologists, and analysts to help them deal with this rapidly evolving method of support and its ethical implications	180	6 (62.5)	5.88 (0.80)	6.0 (6.0–6.0)	Strongly agree
Hurried pathologists will often take “shortcuts” by accepting AI interpretations without verification	171	5 (45.8)	5.08 (1.02)	5.0 (5.0–6.0)	Agree
Potentially-biased algorithms due to lack of demographic diversity in training datasets will lead to diagnostic errors	174	5 (62.5)	5.13 (0.95)	5.0 (5.0–5.5)	Agree
Data inferences that may impact on patient anonymity will lead to ethical issues	178	5 (50.0)	5.17 (0.87)	5.0 (5.0–6.0)	Agree

For the Mode, (%) designates the percentage of panellists who selected that score. AI, artificial intelligence; SD, standard deviation; IQR, interquartile range; CLIA, Clinical Laboratory Improvement Amendments. Item # refers to the question # on the survey questionnaire. Likert scale interpretation: *Agreement level*: 1 = Very strongly disagree, 2 = Strongly disagree, 3 = Disagree, 4 = Neither agree nor disagree, 5 = Agree, 6 = Strongly agree, 7 = Very strongly agree.

### Impact of AI on pathology KPIs

There was agreement that AI would improve multiple laboratory KPIs (Table 1 and Table S4), but that histopathologic analyses would become more quantitative and diagnostic reports more complex. There was also agreement that AI would lead to greater standardization of diagnostic and pre-analytical processes; as a result, an increase in satisfaction of referring physicians was expected. Statements on the likelihood of cost-per-case and number of second-opinion consultations decreasing with AI use failed to reach consensus, although most predicted that cost-per-case would not decrease, at least within the next 8–10 years. Table 1 indicates that, by 2030, there will be growth in CPath as a subspecialty, with AI applications assisting pathologists in making more accurate, standardized, objective, quantitative, and complete diagnoses.

### AI's impact on the pathology workforce and tasks

There was agreement that AI adoption would not greatly affect the size of the overall pathology job market; however, the types and frequencies of tasks performed by pathologists and laboratory technicians were expected to change significantly. It was also agreed that AI would facilitate subspecialisation, with the number of CPathologists greatly increasing (Table 2), and that pathologists would be routinely involved in new tasks related to AI incorporation into their workflows, participating in the development of AI solutions and contributing to the definition of new patient categories. AI adoption was expected to increase pathologist involvement in ancillary activities (research, multidisciplinary conferences, etc.).

Compared to panellists who had been in practice ≥11 years, those in practice ≤10 years more strongly agreed that digital pathologic diagnosis without physical glass slides would be routine by 2030 (*p* = 0.041, Wilcoxon rank-sum test), with median = 7.0 [IQR 6.0–7.0] (compared to 6.0 [IQR 5.0–6.0], for those in practice ≥11 years) and mean = 6.43 [SD (standard deviation) = 0.79], compared to 5.23 [SD = 1.79] for those in practice ≥11 years. The same group also more strongly agreed that interpretation of computationally-derived measurements and evaluations would be routine (*p* = 0.048, Wilcoxon rank-sum test), with median = 7.0 [IQR 6.0–7.0] (compared to 6.0 [6.0–6.0], for those in practice ≥11 years) and mean = 6.71 [SD = 0.49] (compared to 5.82 [SD = 1.19], for those in practice ≥11 years).

The work of pathology technicians was also expected to undergo major changes due to AI adoption (Table 2). Technicians would routinely be involved in digital and computational workflows by operating scanners, calibrating devices, and QA/QC'ing digitized images. A slight majority of panellists thought that technicians might directly participate in AI-assisted diagnosis, although consensus was not reached.

Panellists subspecializing in informatics/digital/CPath more strongly felt that technicians would routinely be providing digital pathology support by performing device calibration and other tasks) (*p* = 0.0050, Wilcoxon rank-sum test), with median = 7.0 [IQR 6.0–7.0] and mean = 6.63 [SD = 0.52] (compared to median = 6.0 [IQR 5.0–6.0] and mean = 5.50 [SD = 1.15] for those not subspecializing in informatics/digital/CPath). Similarly, this group felt more strongly that technicians would routinely be involved in assessing and improving the consistency of histologic preparation to make images more suitable for AI (*p* = 0.049, Wilcoxon rank-sum test), with mean = 6.25 [SD = 0.46] (compared to mean = 5.63 [SD = 0.72], with the same medians of 6.0 [IQR 6.0–6.5 vs 5.0–6.0], for those not subspecializing in informatics/digital/CPath).

### Applications of AI to pathology and integrated diagnostics

AI was expected to positively impact many aspects of the pathology workflow, with several applications expected to be in routine use by 2030 (Table 3). For the analysis and interpretation of histologic images, these included algorithms for identifying hotspots (i.e., during mitotic counts), microorganisms (acid-fast bacilli and *Helicobacter pylori*) and cancer, and tumour grading. There was also certainty that AI would be in routine use for automated quantification of immunohistochemical (IHC) and immunofluorescent (IF) biomarkers, counting of mitotic figures and lymphocytes, and lymph node metastasis identification. Manual tasks expected to be replaced by AI included size measurement and perineural and lymphovascular invasion detection in malignancies. In addition, it was expected that AI-based computational/virtual staining would replace the need for multiplex IHC/IF.

AI was expected to increase diagnostic efficiency by prioritizing regions of interest (ROIs) suspicious for cancer involvement for pathologist review, and by pre-populating diagnostic reports using medical records, gross descriptions, and AI-generated image interpretations. In addition, it was expected to facilitate more accurate diagnoses by importing contextually relevant clinical data for pathologist review, providing a set of differential diagnoses, and prompting second reads on cases with discrepancies between pathologist and AI-rendered diagnoses.

AI was expected to significantly impact laboratory workflows through automated case prioritization and ancillary stain recommendation or ordering (Table 3). Regarding differences in opinion between the panellist subgroups, those subspecializing in informatics/digital/CPath less strongly believed that AI would routinely be used for eosinophil quantification in eosinophilic esophagitis (*p* = 0.049 (Wilcoxon rank-sum test), mean = 5.75 [SD = 0.46]) compared to the other subspecialists (mean = 6.31 [SD = 0.70], with the same medians of 6.0 [IQRs 5.5–6.0 vs 6.0–7.0]).

AI was expected to foster the integration of pathology with other diagnostic modalities (Table 4), with multimodal-AI enabling the combination of diverse data types (gross/macroscopic, microscopic, radiologic, and genomic) in a single interface and facilitating integrated diagnostic reporting for diseases such as prostate cancer. Consequently, it was expected that AI-powered integrated diagnostics would lead to significant advances in personalized healthcare by categorizing patients based on differential risk-stratification (prognostic) roadmaps and clinical outcome predictions.

Panellists with longer practice experience more strongly believed that the integration of pathologic and radiologic data would routinely be used to select patients for active surveillance versus radiotherapy/surgery in prostate cancer (*p* = 0.044 (Wilcoxon rank-sum test), equal medians of 5.0 [IQRs 3.0–5.0 vs 5.0–6.0], mean = 5.29 [SD = 1.10] for ≥11 years' and mean = 4.29 [SD = 1.25] for ≤10 years' practice experience, respectively). They also more strongly believed that AI would routinely be used to build risk stratification roadmaps for patients based on multimodal input data (*p* = 0.020, Wilcoxon rank-sum test; equal medians of 5.0 [IQR 5.0–6.0 vs 4.0–5.0], mean = 5.41 [SD = 0.94] versus 4.43 [SD = 0.79] for ≥11 years' and ≤10 years’ practice experience, respectively).

It was thought likely that AI would not simply assist with, but would fully replace, pathologists on several tasks (Table 5), and that work assignment and case triage were likely to be fully AI-automated.

The panellists practicing outside of North America thought it more likely that colorectal polyp screening would be fully delegated to AI (*p* = 0.047, Wilcoxon rank-sum test; mean = 6.0 [SD = 0.89] and median = 6 [IQR 6.0–7.0] for non-North American panellists; mean = 5.23 [SD = 1.01] and median = 5.0 [IQR 5.0–6.0] for North American panellists). Those in practice longer (≥11 years) thought it more likely that mitotic counts would be fully delegated to AI (*p* = 0.036, Wilcoxon rank-sum test; equal medians of 6.0, mean = 6.29 [SD = 0.69 vs. 5.57 [0.53] for those with ≥11 and ≤ 10 years' practice experience, respectively).

### Regulatory and ethical aspects of AI integration in pathology

The panellists foresaw significant regulatory and ethical challenges posed by AI integration (Table 6) and agreed that both primary diagnostic and secondary (e.g., advisory/assistive) algorithms would have to meet strict regulatory requirements. There was agreement that regulatory bodies would create new guidelines addressing AI integration into pathology, providing specific validation procedures, and simplifying regulatory pathways for AI tools, although clearance of AI software would still be a lengthy and costly process.

There was also agreement that the regulatory approval of adaptive algorithms which continuously evolve in response to new input data would be possible, but that algorithm drift would pose important challenges that would need to be addressed through close post-market surveillance. It was also anticipated that legal disputes would arise regarding liability for diagnostic errors induced by AI, with pathologists still being held legally responsible for AI-assisted diagnoses.

The North American panellists more strongly believed that CLIA regulations and clarification surrounding the use of laboratory data within pathology, as well as laboratory processes, would need to be reviewed and updated (*p* = 0.031, Wilcoxon rank-sum test; median = 6.0 [IQR 6.0–6.0] vs. median = 5.0 [IQR 5.0–6.0] and mean = 6.0 [SD = 0.82] vs. mean = 5.18 [SD = 0.98] for North American vs. other panellists, respectively). Those subspecializing in informatics/digital/CPath less strongly believed that legal disputes would often arise regarding liability for AI-induced diagnostic errors (*p* = 0.018, Wilcoxon rank-sum test; mean = 4.88 [SD = 1.13], median = 5 [4.0–6.0]), compared to those not subspecializing in those areas (mean = 6.06 SD = 0.77], median = 6 [IQR 5.5–7.0]).

It was acknowledged that there would be major ethical issues arising from the full delegation of tasks to AI, such as the likelihood that hurried pathologists would often accept AI interpretations without sufficient verification. Conversely, there was disagreement regarding whether the "black box" nature of AI algorithms would cause pathologists to often make diagnoses without enough clinical explainability. Those in practice fewer years (≤10 years) more strongly believed that ethical issues would result from data inferences which might compromise patient anonymity (*p* = 0.0044, Wilcoxon rank-sum test; mean = 6.0 [SD = 0.82], median = 6.0 [IQR 5.0–7.0]), compared to those in practice ≥11 years (mean = 4.82 [SD = 0.64], median = 5.0 [IQR 4.0–5.0]).

Finally, there was consensus that ethical issues would arise due to: 1) risk for diagnostic error from potentially biased algorithms trained on insufficiently diverse datasets; and 2) lack of proper informed consent when using patient data (which the panel agreed would become a common practice). However, it was expected that regulatory bodies would address the preceding ethical and legal challenges, and that funding bodies would actively promote innovation in AI and medicine, thereby fostering the advancement of AI in pathology. It was also anticipated that AI would be integrated into medical school and continuing medical education curricula in order to help pathologists adapt to this rapidly evolving area and its associated legal and ethical implications.

## Discussion

From this consensus study of 24 experts with first-hand CPath/AI experience, we obtained specific insight into consistently agreed-upon opportunities and challenges, as well as perspectives and predictions, regarding the expected role of AI in pathology over the next decade. Despite the diversity of nationalities, subspecialties, and years of professional experience represented (with all panellists holding attending pathologist and/or faculty positions), the panellists were able to reach consensus agreement on 140 (78.3%) of the 180 items surveyed.

There was particularly strong consensus that AI would improve the KPI of diagnostic accuracy, at least partially by assisting with the detection of rare events (such as small tumour foci and metastases), standardizing the diagnosis and grading of tumours, and making histopathologic analyses more quantitative. There was also particularly strong consensus that the number of specialized CPathologists would greatly increase, as would pathologist involvement in multidisciplinary conferences, and that the types of tasks routinely performed by pathology technicians would change significantly.

It was felt to be *almost certain* that specific pathology AI applications would be routinely used by 2030 (i.e., algorithms for lymph node metastasis identification and mitosis, lymphocyte, and IHC/IF stain quantification). It was also thought *very likely* that algorithms would be routinely used for specific pre-analytical (automated QA/QC, suggestion/ordering of ancillary studies, and case prioritization), analytical (microorganism detection and tumour grading/measurement) and post-analytical tasks (enforcement of mandatory second reads upon significant discrepancies between pathologist and AI-rendered diagnoses). It was felt to be *very likely* that many of these tasks, along with colorectal polyp and cervical cytology screening, case triage/assignment, and contextual electronic health record data lookup, would be *fully delegated* to AI. These predictions are consistent with existing applications in the pathology AI literature.^12^^,^^14^^,^^22^, ^23^, ^24^

Many applications projected to be routinely used by 2030 address basic tasks currently performed by pathologists (in which the ground truth label is typically defined by the pathologist^25^), rather than "aspirational" tasks such as prediction of molecular biomarker status (including gene expression profiles, microsatellite instability, mutational status and copy number alterations), treatment response, survival, and other clinical outcomes directly from morphologic features,^22^^,^^25^ in contrast to the attention paid to these categories by academic researchers and industry stakeholders.^22^^,^^25^, ^26^, ^27^ A recent survey^28^ asking 75 computational pathology experts (with medical and non-medical backgrounds) to rank the degree of interest, importance, and/or promise of 12 solid tumour-specific pathology AI applications revealed that the "aspirational" applications were consistently rated most highly. The somewhat discrepant findings between this and our survey suggest that those with non-medical backgrounds are more optimistic about the near-term role of "aspirational" AI applications.

In a 2018 online survey of pathologists, trainees, and other respondents regarding AI integration into diagnostic pathology,^29^ 81% of respondents predicted AI integration within 5–10 years, 38% felt it would have no impact on pathologist employability, only 42% felt it would create new positions and improve employment prospects, and 20% were concerned or extremely concerned that AI would displace them from their jobs. Approximately 28% were unsure of AI's impact on efficiency or believed that AI would have no or a negative impact on efficiency. In contrast, our panellists were more optimistic regarding the impact of AI on the pathologist workforce, although there was similar reservation regarding whether AI would truly lead to increased efficiency.

Finally, it is worth noting that our panellists could not reach consensus on 39 of 180 statements (Table S4), such as whether AI would reduce the cost-per-case or number of cases requiring pathologist review or increase patient satisfaction. They were uncertain whether AI outputs for clinical decision-making would always need to be reviewed by a pathologist, whether their “black box” nature would cause pathologists to make diagnoses without enough clinical explainability, whether pathologists would make diagnoses contrary to their own judgment because of AI software recommendations, and whether other healthcare professionals could use AI tools to diagnose cases without pathologists. There was also no consensus on whether AI would lead to de-skilling of pathologists^29^ or whether it would be possible to ensure that pathologists took full responsibility for double-checking and confirming AI-rendered diagnoses. Due to the current AI "translation gap" in pathology, there have been a limited number of studies evaluating the impact of AI tools on pathologist behaviour,^11^^,^^12^^,^^30^ laboratory expenditures, medicolegal liability, and patient satisfaction. The lack of consensus regarding these is expected to be resolved as more AI tools are evaluated in prospective clinical settings and more consideration is directed toward ensuring that tools are integrated into workflows in ways that maximize safety, efficiency, and positive patient outcomes.^5^^,^^6^^,^^17^^,^^31^

Similarly, processes for obtaining regulatory approval for AI tools are expected to evolve as the number of vendors seeking to market Cpath/AI algorithms increases. In the United States, the centralized Food and Drug Administration (FDA) is responsible for clearing medical devices (including Cpath/AI algorithms) through one of three pathways: the premarket approval (PMA), the de-novo premarket review, or the 510(k) pathway, depending on the risk level of the device and the availability of a previously-approved predicate device).^32^ As of October 5, 2022, only eight unique AI/ML-enabled medical devices have been cleared by the FDA, of which two are for AP (one for identification of prostate cancer in prostate needle biopsies and the other for Pap smear screening); the remaining 6 tools are all for Hematology (peripheral blood cell counting/analysis).^33^ In contrast to the United States, the regulatory approval process in Europe is decentralized, with Conformité Européenne (CE) mark approval being performed by accredited private Notified Bodies. The number of AI/ML-enabled medical devices with Conformité Européenne (CE) mark approval is greater; from a comprehensive review of devices approved between August 2014 and August 2020,^34^ we identified eight additional AP devices (predominantly in the areas of breast and prostate cancer diagnosis, lymph node metastasis detection, breast immunohistochemistry interpretation, and Ki67 hotspot scoring). In addition to the preceding devices, we were able to identify through an online search 17 more AP devices which had received CE mark approval as of September 2022. We expect the regulatory landscape to evolve as the list of devices and algorithms grows in the coming decade.

This systematic consensus study was subject to a few limitations. Given the voluntary nature and substantial time commitment required to complete all rounds, not all invitees agreed to participate, which could have introduced non-response bias. The inclusion criteria and selection procedure for potential panellists, including use of the PubMed database (which tends to index a larger proportion of English-language publications), could also have led to unintentional geographic bias in our panel, whereby respondents from outside of North America and Europe, such as Asia, Africa, and Latin America, were relatively underrepresented. As a consequence of the location of practice of most of the participants in North America and Europe, our results may or may not be generalizable to other parts of the world. We also note the relative underrepresentation of women within the initial candidate (9 of 39) and final panellist (4 of 24) lists, which likely reflects the general underrepresentation of women in the field of AI.^35^^,^^36^ Both the underrepresentation of women and non-North American/European panellists are important limitations of the current study which we hope will be addressed by future more geographically- and gender-diverse surveys that could be more specifically targeted toward demographics not well-represented in the current one. Lastly, due to the focus of the study on soliciting the opinions of attending pathologist/faculty-level individuals with specific experience in AI/CPath, other potential stakeholders such as pathologists without AI/CPath experience, pathology trainees and technicians, non-pathologist physicians, and patients, were not represented in our panel. It will be important to take into consideration the opinions of these (and other additional) stakeholder groups, as the field of Cpath/AI moves forward.

In conclusion, the results of this systematic consensus study have provided a detailed vision of what pathology might look like in 2030, from the standpoint of those with frontline experience developing and evaluating pathology AI tools. AI is expected to have a deep impact on pathology, and our study provides detailed insight into the current challenges and expectations surrounding its role in pathology, including timely and relevant information regarding how pathology care might be delivered in the future, assuming all regulatory and ethical questions are addressed.^16^^,^^17^^,^^32^ While we expect that our findings will be of great interest to a wide variety of stakeholders, we also hope that the preceding limitations will be sufficiently addressed in forthcoming studies, with our survey and its freely available data collection forms serving as a model for independent validation and extension.

## Contributors

Conceptualization: M.A.B., J.A-F., and J.S.; Methodology: M.A.B.; Investigation: M.A.B., D.S.M., A.B., J.V.L., L.P., J.Y.C., B.D., L.E., C.E., A.B.F., F.F., R.G.M., D.J.H., M.D.H, E.H., K.A.I., A.K., M.K., J.K.L., M.E.S., J.H.S., J.M.T., B.W., J.A-F., and J.S.; Data Curation: M.A.B.; Formal Analysis: M.A.B., J.A-F., and J.S.; Supervision: C.C-S., V.S-T., A.L., J.A-F., and J.S.; Writing - original draft: M.A.B., D.S.M., J.A-F., and J.S.; Writing - review & editing: D.S.M, A.B., J.V.L., L.P., J.Y.C., B.D., L.E., C.E., A.B.F., F.F., R.G.M., D.J.H., M.D.H, E.H., K.A.I., A.K., M.K., J.K.L., M.E.S., J.H.S., M.T., B.W., and J.S.; Project administration: M.A.B., J.A-F., and J.S. The corresponding authors, M.A.B. and J.S., verified all underlying data and took final responsibility for the decision to submit for publication. All authors read and approved the final submitted manuscript.

## Data sharing statement

The full survey questionnaire and de-identified raw and aggregate participant results are available in the Appendix and supplementary materials accompanying this manuscript.

## Declaration of interests

M.A.B. is a board member of Cells IA Technologies; D.S.M. received consulting fees from, and is a scientific advisory board member of, Epredia and 10.13039/100004337Roche, received honoraria for a sponsored presentation from Roche, and holds a leadership or fiduciary role in the Digital Pathology Association (DPA); J.V.L. received research funding from ContextVision, Sectra, and 10.13039/100004320Philips, consulting fees from, and is a scientific advisory board member of, ContextVision and Philips, is a member of the Board of Directors of the DPA, Chair of the AI Taskforce of the European Society of Pathology, and is Chief Scientific Officer of, and holds stocks or stock options from, Aiosyn B.V.; L.P. received consulting fees from Hamamatsu and Ibex, has patents planned, issued or pending (LeanAP Innovators), holds an unpaid leadership or fiduciary role in other board, society, committee or advocacy group (DPA and ASC), and is a shareholder of Ibex; C.E. received consulting fees from Mindpeak, payment or honoraria for lectures, presentations, speakers bureaus, manuscript writing or educational events from 10.13039/501100018828Leica and 3DHISTECH, and payment for expert testimony from MSD; D.J.H received royalties from Up-To-Date/LWW for the creation of educational content, consulting fees from IQVIA/Genae and VitaDx, and is a board member and shareholder of Techcyte Inc.; M.D.H received research funding from the 10.13039/100000054National Cancer Institute (NCI), 10.13039/100000002National Institutes of Health (NIH), and support for attending meetings and/or travel from the College of American Pathologists (CAP), DPA, and European Society for Digital and Integrative Pathology, and holds an unpaid leadership or fiduciary role in the DPA; M.E.S. and is a board member and shareholder of Techcyte Inc.; B.W. received honoraria for presentations from Leica Biosystems and is a scientific advisory board member of Paige AI; A.L. received honoraria from General Electric for lectures, and is a board member of Siemens Healthineers and Cells IA Technologies; J.A.F. is a shareholder of Cells IA Technologies; J.S. received institutional research funding from Google/Alphabet Inc. and 10.13039/100019703Lunit Inc., consulting fees from KCK MedTech, and is an advisory board member of Crosscope, Inc. The remaining authors declare no competing interests.

## Appendix A Supplementary data

Supplementary Fig. S1 and Tables S1–S4

Copy of Delphi Round 2

Raw data_Delphi Round 1 (Responses)_anon

Raw data_Delphi Round 2 (Responses)_anon

Raw data_Delphi Round 3 (Responses)_anon
