# Supplementary material for: Computational pathology in 2030: a Delphi study forecasting the role of AI in pathology within the next decade
Source: eBioMedicine. 2023 Jan 4;88:104427. doi: 10.1016/j.ebiom.2022.104427 (PMC9823157; doi:10.1016/j.ebiom.2022.104427)
Supplement: Supplementary Fig. S1 and Tables S1–S4 [file mmc1.docx]

**Appendix for:**

**Computational pathology in 2030: A Delphi study forecasting the role of AI in pathology within the next decade**

M Alvaro Berbís1,2,* David S. McClintock3, Andrey Bychkov4, Jeroen Van der Laak5, Liron Pantanowitz6, Jochen K Lennerz7, Jerome Y Cheng6, Brett Delahunt8, Lars Egevad9, Catarina Eloy10, Alton B Farris III11, Filippo Fraggetta12, Raimundo García del Moral13, Douglas J. Hartman14, Markus D Herrmann15, Eva Hollemans16, Kenneth A Iczkowski17, Aly Karsan18, Mark Kriegsmann19, Mohamed E. Salama20, John H. Sinard21, J. Mark Tuthill22, Bethany Williams23, César Casado-Sánchez24, Víctor Sánchez-Turrión25, Antonio Luna26, José Aneiros-Fernández1,12, Jeanne Shen27*

**Table of contents Page**

1. **Supplementary Figures 2**
2. **Supplementary Tables 3**

**Supplementary Figures**

**
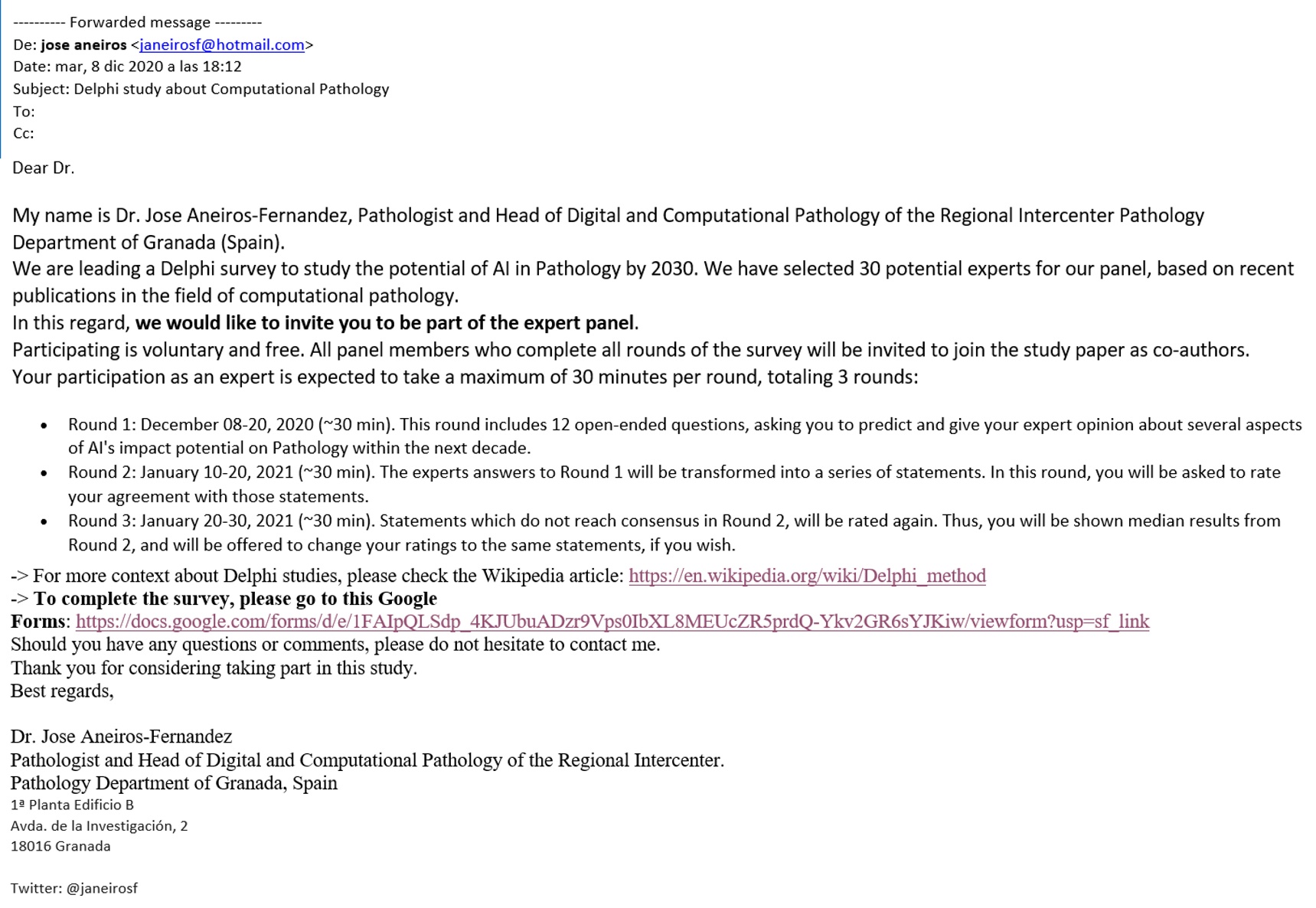
**

**Figure S1. Invitation email sent by Dr. Aneiros-Fernández to all candidates selected as potential participants in the reported Delphi study.**

**Supplementary Tables**

**Table S1: Demographics of the expert panel (n=24)**

| **Categories** | **n (%)** |
| --- | --- |
| *Country of residence* | |
| United States of America | 12 (50%) |
| The Netherlands | 2 (8·3%) |
| Spain | 2 (8·3%) |
| United Kingdom | 1 (4·2%) |
| Germany | 1 (4·2%) |
| Italy | 1 (4·2%) |
| Sweden | 1 (4·2%) |
| Portugal | 1 (4·2%) |
| Canada | 1 (4·2%) |
| Japan | 1 (4·2%) |
| New Zealand | 1 (4·2%) |
| *Area of subspecialization (multiple responses possible)* | |
| Informatics, computational pathology, digital pathology | 8 (33·3%) |
| Hematopathology | 4 (16·7%) |
| Genitourinary pathology | 3 (12·5%) |
| Gastrointestinal pathology | 3 (12·5%) |
| Endocrine pathology | 2 (8·3%) |
| Dermatopathology | 2 (8·3%) |
| Surgical pathology | 2 (8·3%) |
| Molecular pathology | 2 (8·3%) |
| Thoracic pathology | 1 (4·2%) |
| Bone & soft tissue pathology | 1 (4·2%) |
| *Years of experience* | |
| 0–10 | 7 (29·2%) |
| 11–20 | 8 (33·3%) |
| 21–30 | 7 (29·2%) |
| 31–40 | 2 (8·3%) |
| *Gender* | |
| Female | 4 (16·7%) |
| Male | 20 (83·3%) |

**Table S2:** Round 1 Questions

| **Section 1: Forecasts about the future** (please answer according to what you believe will happen by 2030, instead of what you would like to see happen) | |
| --- | --- |
| 1 | On what key performance indicators related to pathology do you believe AI will have a positive impact? |
| 2 | How do you think AI will impact the pathology workforce (jobs which will be created and jobs which will be destroyed) by 2030? |
| 3 | What new tasks will pathologists be involved in? |
| 4 | What new tasks will pathology technicians be involved in, or what existing tasks will they take on more responsibility for? |
| 5 | Which tasks currently performed by pathologists will be fully automated by AI by 2030? |
| **Section 2: Applications of AI in pathology** (please cite any existing or potential AI-based tools which, in your opinion, would bring value to pathologists. Be as specific as possible) | |
| 6 | In what ways can AI be used to improve diagnostic precision? |
| 7 | In what ways can AI be used to speed up or facilitate the work of pathologists? |
| 8 | What examples of AI tools or applications would bring value to the analysis and interpretation of histological images? |
| 9 | What examples of AI tools or applications would bring value to other aspects of the laboratory workflow? |
| 10 | In what ways can AI be used to bring value to integrated diagnostics? (integrated diagnostics refers to the convergence of two or more diagnostic techniques, such as pathology, radiology, genomics) |
| **Section 3: Ethical and regulatory aspects** | |
| 11 | What regulatory challenges will have to be overcome for the generalized adoption of AI in the pathology setting? |
| 12 | What ethical issues could arise from the use (and potential misuse) of AI in the pathology setting? |

AI, artificial intelligence.

**Table S3.**  Likert scales for the different sections (7-point)

| **Point Score** | **Agreement scale** | **Likelihood scale** | **Job number variation scale** | **Involvement scale** |
| --- | --- | --- | --- | --- |
| 1 | Very strongly disagree | Impossible | Dramatically decrease | Not involved at all |
| 2 | Strongly disagree | Very unlikely | Greatly decrease | Rarely |
| 3 | Disagree | Unlikely | Somewhat decrease | Somewhat |
| 4 | Neither agree nor disagree | Even chance / neutral | Remain the same | Sometimes |
| 5 | Agree | Likely | Somewhat increase | Often |
| 6 | Strongly agree | Very likely | Greatly increase | Routine |
| 7 | Very strongly agree | Certain | Dramatically increase | Daily |

**Table S4.** Consensus results after Round 3

| **Statement** | **Mode (%)** | **Mean (SD)** | **Median** | **IQR** | **Result** |
| --- | --- | --- | --- | --- | --- |
| **Section 1: Impact of AI in Pathology KPIs** | | | | | |
| *By 2030, due to the integration of AI in the pathology setting...* | | | | | |
| 1. Time required per case will decrease | 5 (41·7) | 4.71 (0·95) | 5·0 | 1·0 | Agree |
| 2. Cost per case will decrease | 4 (33·3) | 3.33 (1·31) | 3·0 | 1.5 | No consensus |
| 3. Standardization of pre-analytical processes (staining and slicing techniques) will increase | 5 (41·7) | 5.38 (0·92) | 5·0 | 1·0 | Agree |
| 4. The number of unnecessary IHC stains performed will decrease | 5 (45·8) | 4.50 (1·02) | 5·0 | 1·0 | Agree |
| 5. Pathologists will look at a substantially lower number of cases, since cases will be filtered (such as negative biopsies) | 5 (33·3) | 4.33 (1·46) | 5·0 | 2·0 | No consensus |
| 6. Diagnostic accuracy will increase | 6 (58·3) | 5.67 (1·05) | 6·0 | 1·0 | Strongly agree |
| 7. Diagnosis and grading of tumors will be more standardized, bringing more objectivity to the diagnosis of certain entities that are currently subject to high interobserver variability | 6 (62·5) | 6.04 (0·62) | 6·0 | 0·0 | Strongly agree |
| 8. Detection of rare events (small metastases, small tumor foci) will increase | 6 (62·5) | 5.88 (1·03) | 6·0 | 0·0 | Strongly agree |
| 9. Analyses will be more quantitative | 6 (45·8) | 6.21 (0·72) | 6·0 | 1·0 | Strongly agree |
| 10. Completeness of reports will increase | 5 (54·2) | 5.13 (1·03) | 5·0 | 1·0 | Agree |
| 11. Complexity of reports will increase | 5 (50·0) | 5.13 (1·12) | 5·0 | 1·0 | Agree |
| 12. Quality of reports will increase | 5 (33·3) | 5.38 (1·24) | 5·0 | 1·0 | Agree |
| 13. The number of second-opinion consultations will decrease | 4 (37·5) | 3.58 (0·93) | 4·0 | 1·0 | Neither agree nor disagree |
| 14. Satisfaction of referring physicians will increase | 5 (50·0) | 4.67 (1·17) | 5·0 | 1·0 | Agree |
| 15. Patient satisfaction will increase | 5 (37·5) | 4.75 (1·03) | 5·0 | 1.5 | No consensus |
| **Section 2: Impact of AI on the Pathology workforce** | | | | | |
| *By 2030, due to the integration of AI in the pathology setting...* | | | | | |
| 16. The number of jobs for pathologists will | 4 (66·7) | 3.88 (0·68) | 4·0 | 0·0 | Remain the same |
| 17. The number of jobs for pathology technicians will | 5 (54·2) | 4.63 (0·97) | 5·0 | 1·0 | Somewhat increase |
| 18. The number of jobs for IT staff will | 5 (50·0) | 5.54 (0·93) | 5·0 | 1·0 | Somewhat increase |
| 19. The number of jobs in administrative positions will | 4 (54·2) | 4.04 (0·86) | 4·0 | 0·5 | Remain the same |
| 20. The ratio of general pathologists will | 4 (50·0) | 3.79 (0·93) | 4·0 | 1·0 | Remain the same |
| 21. The ratio of subspecialized pathologists will | 5 (41·7) | 4.63 (0·92) | 5·0 | 1·0 | Somewhat increase |
| 22. The number of specialized "computational" pathologists will | 6 (45·8) | 5.75 (0·79) | 6·0 | 1·0 | Greatly increase |
| 23. The number of overall jobs in Pathology will | 5 (41·7) | 4.38 (0·92) | 4·0 | 1·0 | Remain the same |
| 24. Beyond 2030 (by 2040), the number of jobs for pathologists will | 4 (50·0) | 3.79 (0·78) | 4·0 | 1·0 | Remain the same |
| 25. Beyond 2030 (by 2040), the overall number of jobs in Pathology will | 5 (45·8) | 4.25 (1·03) | 4.5 | 1·0 | Somewhat increase |
|  | **Mode (%)** | **Mean (SD)** | **Median** | **IQR** | **Result** |
| 26. Practices with retiring pathologists may choose not to replace them | 5 (37·5) | 4.42 (1·28) | 5·0 | 2·0 | No consensus |
| 27. AI will pose an increased incentive to retire, for those pathologists who are unwilling to apply AI to their daily job | 5 (50·0) | 4.54 (1·14) | 5·0 | 1·5 | No consensus |
| 28. Pathology will attract more and better talent into the specialty, because of an increased interest due to AI developments in the field | 5 (45·8) | 5.04 (0·91) | 5·0 | 1·5 | No consensus |
| **Section 3: Tasks of pathologists** | | | | | |
| *By 2030, due to the integration of AI in the pathology setting, the degree of involvement of pathologists in these tasks will be…* | | | | | |
| 29. Digital pathologic diagnosis without the use of physical glass slides | 6 (50·0) | 5.58 (1·64) | 6·0 | 1·0 | Routine |
| 30. Interpretation of computationally derived measurements and evaluations | 6 (45·8) | 6.08 (1·10) | 6·0 | 1·0 | Routine |
| 31. Collaboration with EHR teams regarding the use of laboratory data for a wide range of clinical decision support tools | 6 (45·8) | 5.25 (1·03) | 5.5 | 1·0 | Routine |
| 32. Direct patient care activities | 4 (45·8) | 4.13 (1·33) | 4·0 | 1·0 | Sometimes |
| 33. Decision-making related to patient treatment | 5 (62·5) | 4.79 (1·18) | 5·0 | 0·5 | Often |
| 34. Patient identification for clinical trials | 5 (45·8) | 4.88 (1·36) | 5·0 | 1·0 | Often |
| 35. Evaluating different kinds of AI software and deciding whether these are appropriate for their workflow | 6 (62·5) | 5.54 (1·14) | 6·0 | 1·0 | Routine |
| 36. Validation and QA/QC of AI solutions | 6 (58·3) | 5.63 (1·13) | 6·0 | 1·0 | Routine |
| 37. Validation and QA/QC of AI-rendered diagnoses | 6 (50·0) | 5.88 (1·23) | 6·0 | 1·0 | Routine |
| 38. Defining new categories of patients, based on new data made available through AI | 5 (41·7) | 5.04 (1·43) | 5·0 | 1·0 | Often |
| 39. Design of AI solutions | 5 (45·8) | 4.63 (0·88) | 5·0 | 1·0 | Often |
| 40. Development of AI solutions, including annotation of image data and algorithm training | 5 (58·3) | 4.75 (0·79) | 5·0 | 1·0 | Often |
| 41. Mass spectrometry analysis | 3 (37·5) | 3.33 (1·31) | 3·0 | 1·5 | No consensus |
| 42. Molecular pathology, including NGS analysis | 6 (33·3) | 5.38 (1·35) | 6·0 | 1·5 | No consensus |
| 43. In situ genetic analysis using multiplex technology | 5 (33·3) | 4.88 (1·03) | 5·0 | 2·0 | No consensus |
| 44. Pathologists will be more involved in diagnostic tumor boards | 6 (54·2) | 5.58 (1·06) | 6·0 | 1·0 | Strongly agree |
| 45 Pathologists will be more involved in multidisciplinary conferences | 6 (58·3) | 5.63 (1·06) | 6·0 | 1·0 | Strongly agree |
| 46. Pathologists will be more involved in research activities | 5 (37·5) | 5.42 (1·06) | 5·0 | 1·0 | Agree |
| 47. Pathologists will be spending more time in the study of rare lesions | 5 (45·8) | 5.13 (1·03) | 5·0 | 1·0 | Agree |
| **Section 4: Tasks of pathology technicians** | | | | | |
| *By 2030, due to the integration of AI in the pathology setting, the degree of involvement of pathology technicians in these tasks will be…* | | | | | |
| 48. Operation of digital slide scanners, digitization and image management | 7 (58·3) | 6.25 (1·22) | 7·0 | 1·0 | Daily |
| 49. QA/QC of digitized images | 7 (50·0) | 6.08 (1·41) | 6.5 | 1·0 | Daily |
| 50. Digital Pathology support for pathologists and other users, such as device calibration | 6 (54·2) | 5.88 (1·12) | 6·0 | 0·5 | Routine |
|  | **Mode (%)** | **Mean (SD)** | **Median** | **IQR** | **Result** |
| 51. Assessing histology consistency, i.e. re-addressing SOPs to make slides and corresponding images more suitable for AI (more consistent tissue and staining quality) | 6 (62·5) | 5.83 (0·70) | 6·0 | 0·5 | Routine |
| 52. Sample grossing guided by AI-derived image analysis | 5 (41·7) | 4.13 (0·95) | 4·0 | 1·0 | Sometimes |
| 53. Diagnosis of cervical cancer aided by AI | 6 (54·2) | 5.46 (1·32) | 6·0 | 2·0 | No consensus |
| 54. Diagnosis of other diseases, aided by AI | 5 (33·3) | 4.75 (1·33) | 5·0 | 2·0 | No consensus |
| 55. Counting of quantitative markers, such as Ki-67 | 6 (37·5) | 5.79 (0·93) | 6·0 | 1·5 | No consensus |
| 56. Validation and QA/QC of AI-rendered diagnoses | 5 (45·8) | 5.17 (0·96) | 5·0 | 1·0 | Often |
| 57. Development of AI solutions, including annotation of image data and algorithm training | 4 (37·5) | 4.25 (1·19) | 4·0 | 1·0 | Sometimes |
| 58. Preparation of reports | 5 (33·3) | 4.83 (1·40) | 5·0 | 2·0 | No consensus |
| **Section 5: AI applications in Pathology** | | | | | |
| *By 2030, the probability of these AI tools being used routinely in pathology labs is:* | | | | | |
| 59. AI-assisted laboratory workflow management, including workload assignments to pathologists, residents, and technicians | 5 (45·8) | 5.33 (1·31) | 5·0 | 1·0 | Likely |
| 60. Automatic QA/QC of macroscopic images and grossing | 5 (29·2) | 4.63 (1·28) | 5·0 | 2·0 | No consensus |
| 61. Automated ordering of IHC for specific applications / assisting with selection of immunohistochemical stains needed | 6 (45·8) | 5.46 (0·93) | 6·0 | 1·0 | Very likely |
| 62. Automated QA/QC of IHC positive and negative controls | 6 (54·2) | 5.75 (0·90) | 6·0 | 1·0 | Very likely |
| 63. Pre-selection of potentially cancer-positive samples for pathologist's review, while the bulk of clearly negative samples can be automatically processed | 5 (45·8) | 5.13 (1·26) | 5·0 | 1·0 | Likely |
| 64. Triaging of cases to the most appropriate pathologist at the earliest possible time | 5 (41·7) | 5.08 (1·41) | 5·0 | 1·0 | Likely |
| 65. Generation of H&E-style WSI directly from fresh tissue (bypassing the need for frozen section slide creation) | 5 (33·3) | 4.67 (1·27) | 5·0 | 1·5 | No consensus |
| 66. Up-front case classification associated with IHC selection and followed by triaging to the specialist | 5 (45·8) | 4.83 (1·61) | 5·0 | 1·0 | Likely |
| 67. Computer handling of routine specimens, thus freeing up time for pathologists to handle difficult cases | 5 (50·0) | 4.67 (1·43) | 5·0 | 1·0 | Likely |
| 68. Proposing specific IHC or other molecular methods to solve a specific diagnostic problem | 6 (41·7) | 5.17 (1·34) | 5.5 | 1·0 | Very likely |
| 69. Prioritization of cases (such as cases with neoplasia and infectious organisms in immunosuppressed patients) | 6 (45·8) | 5.50 (1·10) | 6·0 | 1·0 | Very likely |
| 70. Generation of gross descriptions of pathology specimens | 3 (33·3) | 3.92 (1·28) | 4·0 | 2·0 | No consensus |
| 71. Identification of mislabeled specimens (e.g. a prostate biopsy case containing tissue from a different organ) | 5 (45·8) | 5.04 (1·04) | 5·0 | 1·5 | No consensus |
| 72. Automated routing of cases for workup by robotics | 4 (33·3) | 4.17 (1·24) | 4·0 | 2·0 | No consensus |
| 73. Quality control of whole-slide images (scanning process), and detection of poor-quality slides (tissue folds, poor staining). | 6 (66·7) | 6.13 (0·68) | 6·0 | 0·5 | Very likely |
| 74. Quality improvement of whole-slide images | 6 (62·5) | 6.00 (0·93) | 6·0 | 0·5 | Very likely |
| 75. Tools that bring up similar cases with their respective diagnoses for feature comparison | 5 (41·7) | 5.17 (1·05) | 5·0 | 1·5 | No consensus |
|  | **Mode (%)** | **Mean (SD)** | **Median** | **IQR** | **Result** |
| 76. Pre-selecting regions of interest suspicious for cancer for pathologists to view | 7 (45·8) | 6.29 (0·75) | 6·0 | 1·0 | Very likely |
| 77. Identification of hotspot areas | 7 (45·8) | 6.25 (0·85) | 6·0 | 1·0 | Very likely |
| 78. Identification of micrometastases | 7 (50·0) | 6.17 (1·09) | 6.5 | 1·0 | Certain |
| 79. Detection of lymph node metastases | 7 (54·2) | 6.33 (0·87) | 7·0 | 1·0 | Certain |
| 80. Detection of signet ring-cell cancer | 5 (41·7) | 5.29 (1·08) | 5·0 | 1·0 | Likely |
| 81. Detection of microorganisms (AFB, *H. pylori*) | 6 (58·3) | 6.17 (0·87) | 6·0 | 1·0 | Very likely |
| 82. Assisting with tumor grading | 6 (62·5) | 6.21 (0·59) | 6·0 | 1·0 | Very likely |
| 83. Identification of molecular biomarker status directly from H&E WSI: automated mutation detection in cancer, such as non-small cell lung cancer | 5 (37·5) | 4.71 (1·46) | 5·0 | 2·0 | No consensus |
| 84. Identification of molecular biomarker status directly from H&E WSI: prediction of clinical outcome and response to treatment, e.g. in colorectal cancer | 4 (37·5) | 4.42 (1·10) | 4·0 | 1·0 | Even Chance / Neutral |
| 85. Quantification of IHC or IF stains, such as Ki-67, ER, PgR, PD-L1 | 7 (70·8) | 6.67 (0·56) | 7·0 | 1·0 | Certain |
| 86. Quantification of number of mitoses in H&E-stained images | 7 (50·0) | 6.33 (0·76) | 6.5 | 1·0 | Certain |
| 87. Counting lymphocytes | 7 (50·0) | 6.42 (0·65) | 6.5 | 1·0 | Certain |
| 88. Quantification of eosinophils in eosinophilic esophagitis | 6 (62·5) | 6.13 (0·68) | 6·0 | 1·0 | Very likely |
| 89. Quantitation of features (e.g., fibrosis in various organs, liver steatosis, etc.) | 6 (62·5) | 6.29 (0·55) | 6·0 | 1·0 | Very likely |
| 90. Marking of perineural invasion, lymphovascular invasion | 6 (50·0) | 5.79 (0·98) | 6·0 | 1·0 | Very likely |
| 91. Allowing for computational staining instead of multiplexing | 5 (54·2) | 4.67 (1·24) | 5·0 | 1·0 | Likely |
| 92. Providing a set of differential diagnoses on difficult cases | 5 (45·8) | 5.13 (0·90) | 5·0 | 1·0 | Likely |
| 93. Proposing specific additional tests for solving a diagnostic problem (e.g., AI algorithm suggesting STAT6 immunostaining on a spindle cell neoplasm of the pleura) | 6 (37·5) | 5.17 (1·24) | 5·0 | 1·0 | Likely |
| 94. Automated measurements (e.g., of tumor areas) | 6 (54·2) | 6.21 (0·66) | 6·0 | 1·0 | Very likely |
| 95. Ensuring all diagnostically relevant areas on the slide are viewed prior to report finalization | 6 (50·0) | 5.42 (0·83) | 6·0 | 1·0 | Very likely |
| 96. Import of contextually-related data on a case for quick review by the pathologist during diagnostic slide review | 5 (58·3) | 5.21 (0·72) | 5·0 | 1·0 | Likely |
| 97. Mandatory second reads when the pathologist diagnosis does not match the potential AI diagnosis (within a predefined range/percentage; e.g., if the AI tool detects potential tumor on a biopsy but the pathologist reads the biopsy as no evidence of tumor) | 6 (54·2) | 5.79 (0·83) | 6·0 | 1·0 | Very likely |
| 98. Standardization of pathology reports | 6 (66·7) | 5.88 (0·68) | 6·0 | 0·0 | Very likely |
| 99. Pre-populating relevant report details from the medical record/gross description | 5 (54·2) | 5.29 (0·95) | 5·0 | 1·0 | Likely |
| 100. Selection of the appropriate synoptic report based on prior pathology findings, including the current case gross report | 5 (50·0) | 5.38 (0·88) | 5·0 | 1·0 | Likely |
|  | **Mode (%)** | **Mean (SD)** | **Median** | **IQR** | **Result** |
| 101. Pre-populating reports based on AI interpretation of images | 5 (45·8) | 5.13 (1·03) | 5·0 | 1·0 | Likely |
| 102. Finding the source of contaminants | 5 (58·3) | 5.17 (0·96) | 5·0 | 0·5 | Likely |
| **Section 6: Role of AI in integrated diagnostics** | | | | | |
| *By 2030, the probability of these integrated diagnostic applications being used routinely is:* | | | | | |
| 103. Comparison of tumor extent in slides and radiological images | 5 (66·7) | 4.71 (1·00) | 5·0 | 0·0 | Likely |
| 104. Identification of histologic regions to be sampled for genomic testing | 5 (45·8) | 5.38 (1·13) | 5·0 | 1·0 | Likely |
| 105. Prompting of further genetic testing based on AI analysis of histology | 5 (45·8) | 5.13 (0·90) | 5·0 | 1·5 | No consensus |
| 106. Assessment of staging based on combined pathology/genetic data | 5 (45·8) | 4.79 (1·06) | 5·0 | 1·5 | No consensus |
| 107. Correlation of morphological and genomic information in order to interpret genetic aberrations found | 5 (66·7) | 4.92 (0·97) | 5·0 | 0·0 | Likely |
| 108. Extraction of molecular data from radiological or histological images | 5 (37·5) | 4.58 (1·25) | 5·0 | 1·5 | No consensus |
| 109. Prediction of biomarker status and clinical outcomes for personalized medicine, based on integrated diagnostics | 5 (58·3) | 5.08 (1·14) | 5·0 | 0·5 | Likely |
| 110. Identifying discrepancies between radiologic and pathologic diagnoses | 5 (62·5) | 4.71 (1·12) | 5·0 | 0·0 | Likely |
| 111. Algorithms for interpretation of tumor treatment response in radiology, based on pathology data | 5 (45·8) | 4.42 (1·10) | 5·0 | 1·0 | Likely |
| 112. Algorithms that transfer pathology diagnostic information to radiologic images: Based on the pathology diagnosis, a heatmap is shown on radiology images, displaying tumor magnitudes | 4 (41·7) | 4.17 (1·05) | 4·0 | 1·0 | Even Chance / Neutral |
| 113. Combination of all data (macro, micro, radiology, genomic) on the same screen | 5 (50·0) | 4.79 (1·38) | 5·0 | 1·0 | Likely |
| 114. Diagnoses based on combination of genomics, radiomics and multiparameter IHC along with blood counts and serum analysis | 5 (37·5) | 4.21 (1·06) | 4·0 | 2·0 | No consensus |
| 115. Displaying a list of the most probable diagnoses for a particular case, according to the combination of different diagnostic techniques | 4 (37·5) | 4.33 (1·13) | 4·0 | 1·0 | Even Chance / Neutral |
| 116. Reduction of healthcare costs by avoiding redundancy of testing | 5 (41·7) | 4.50 (1·14) | 5·0 | 1·0 | Likely |
| 117. Clinical use of prediction models resulting from multivariable analysis of data from different modalities (pathology, radiology, genetics) | 5 (41·7) | 5.08 (1·06) | 5·0 | 1·5 | No consensus |
| 118. Selection of patients with prostate cancer for active surveillance versus radiotherapy/surgery, based on integration of pathology and radiology data | 5 (54·2) | 5.00 (1·22) | 5·0 | 1·0 | Likely |
| 119. Creation of new categories of patients by integrating all "big data" from pathology, clinical lab, radiology, and genomics | 5 (58·3) | 5.04 (1·16) | 5·0 | 0·0 | Likely |
| 120. Building risk stratification (prognostic) roadmaps for individual patients based on input from histology, radiology, and genomics | 5 (54·2) | 5.13 (0·99) | 5·0 | 1·0 | Likely |
| 121. Use of integrated reports for select conditions, e.g., prostate cancer | 5 (33·3) | 5.33 (1·31) | 5·0 | 1·0 | Likely |
|  | **Mode (%)** | **Mean (SD)** | **Median** | **IQR** | **Result** |
| **Section 7: Pathology tasks fully automated by AI in 2030** | | | | | |
| *By 2030, the probability of these tasks being fully delegated to AI in pathology labs is...* | | | | | |
| 122. Screening of tissues with a cancer diagnosis to select regions for tissue coring or macroscopic dissection | 5 (58·3) | 5.08 (1·02) | 5·0 | 0·5 | Likely |
| 123. Selection of which IHC to be performed | 5 (41·7) | 4.54 (1·35) | 5·0 | 1·0 | Likely |
| 124. Verification of positive and negative controls for IHC | 6 (58·3) | 5.71 (0·91) | 6·0 | 1·0 | Very likely |
| 125. Prioritization of cases | 6 (50·0) | 5.54 (1·47) | 6·0 | 1·0 | Very likely |
| 126. Triage of cases to appropriate pathologists | 6 (45·8) | 5.46 (1·25) | 6·0 | 1·0 | Very likely |
| 127. Contextual data lookup on patients from the EHR relevant to the pathology case being reviewed | 6 (50·0) | 5.25 (1·15) | 6·0 | 1·0 | Very likely |
| 128. Slide QC (e.g., detection of tissue folds and tears, stain quality evaluation, etc.) | 6 (58·3) | 5.88 (1·03) | 6·0 | 0·0 | Very likely |
| 129. Screening of microorganisms, such as AFB and *H. pylori* | 6 (58·3) | 5.96 (0·75) | 6·0 | 0·0 | Very likely |
| 130. Screening of colorectal polyps | 6 (41·7) | 5.58 (1·02) | 6·0 | 1·0 | Very likely |
| 131. Cervical cytology screening | 7 (41·7) | 6.21 (0·78) | 6·0 | 1·0 | Very likely |
| 132. Screening lymph nodes for metastases | 6 (54·2) | 5.83 (0·76) | 6·0 | 1·0 | Very likely |
| 133. Detection of tumors in H&E-stained WSI | 5 (37·5) | 5.04 (1·20) | 5·0 | 2·0 | No consensus |
| 134. Slide screening for regions of interest | 5 (50·0) | 5.13 (0·99) | 5·0 | 1·0 | Likely |
| 135. Measurement tasks | 6 , 7 (41·7) | 6.17 (0·92) | 6·0 | 1·0 | Very likely |
| 136. Assessing tumor cellularity | 6 (33·3) | 5.83 (0·96) | 6·0 | 2·0 | No consensus |
| 137. Quantification of IHC or IF stains, such as Ki-67, ER, PgR, PD-L1 | 6 (45·8) | 6.29 (0·69) | 6·0 | 1·0 | Very likely |
| 138. Quantification of mitotic count on H&E-stained images | 6 (50·0) | 6.08 (0·72) | 6·0 | 1·0 | Very likely |
| 139. Bone marrow differential counts | 6 (37·5) | 5.54 (1·02) | 6·0 | 1·0 | Very likely |
| 140. Classification of skin lesions | 5 (37·5) | 4.58 (1·18) | 5·0 | 1·0 | Likely |
| 141. MIB-1 scoring | 6 (54·2) | 6.04 (0·91) | 6·0 | 1·0 | Very likely |
| 142. Grading of dysplasia | 6 (29·2) | 4.88 (1·26) | 5·0 | 2·0 | No consensus |
| 143. Assessing extent of liver steatosis and fibrosis | 6 (41·7) | 5.54 (1·14) | 6·0 | 1·0 | Very likely |
| 144. Grading of prostate cancer | 6 (29·2) | 5.54 (1·18) | 6·0 | 1·5 | No consensus |
| 145. Grading of breast cancer | 5 (33·3) | 5.42 (1·14) | 5·0 | 1·0 | Likely |
| 146. Grading of colorectal cancer | 5 (37·5) | 5.33 (1·09) | 5·0 | 1·0 | Likely |
| 147. Grading of lung cancer | 5 (41·7) | 5.17 (1·05) | 5·0 | 1·5 | No consensus |
| 148. Integration of data from multiple IHC stains | 5 , 6 (33·3) | 5.13 (1·15) | 5·0 | 1·5 | No consensus |
| 149. Correlation of morphologic, immunofluorescence and molecular pathology images | 3 , 4 (25·0) | 4.42 (1·32) | 4·0 | 2·5 | No consensus |
|  | **Mode (%)** | **Mean (SD)** | **Median** | **IQR** | **Result** |
| **Section 8: Regulatory aspects** | | | | | |
| *By 2030, regarding the integration of AI in pathology...* | | | | | |
| 150. A set of new guidelines will be developed, specifically addressing the integration of AI in pathology | 7 (79·2) | 6.63 (0·82) | 7·0 | 0·0 | Very strongly agree |
| 151. Specific validation procedures for different types of AI tools will be defined by regulatory bodies | 7 (58·3) | 6.46 (0·72) | 7·0 | 1·0 | Very strongly agree |
| 152. Regulatory pathways concerning AI tools for pathology will be simplified | 5 (37·5) | 4.54 (1·32) | 5·0 | 1·0 | Agree |
| 153. Approving developers once, circumventing the need to approve every app developed by a cleared AI company, will be possible | 4 (33·3) | 4.04 (1·16) | 4·0 | 2·0 | No consensus |
| 154. Validation of families of algorithms with the same basic structure, rather than on an individual basis for each algorithm and application, will be possible | 5 (41·7) | 4.25 (0·99) | 4·0 | 1·0 | Neither agree nor disagree |
| 155. Approving adaptive algorithms that constantly change will be possible | 5 (45·8) | 4.42 (1·18) | 5·0 | 1·0 | Agree |
| 156. Any legal and administrative barriers to use of anonymized images in education and research will be overcome | 5 (41·7) | 4.46 (1·14) | 5·0 | 1·5 | No consensus |
| 157. Meeting regulatory requirements for most AI applications will be a lengthy and costly process, as it will involve large-scale prospective studies | 5 (37·5) | 5.46 (1·25) | 5.5 | 1·0 | Strongly agree |
| 158. Definition of endpoints for clinical validation studies will be a common problem | 6 (37·5) | 5.50 (1·14) | 6·0 | 1·0 | Strongly agree |
| 159. Post-marketing surveillance will pose important challenges, due to algorithm drift | 6 (41·7) | 5.50 (1·06) | 6·0 | 1·0 | Strongly agree |
| 160. Regulatory issues will not be a challenge for AI use in pathology, since the medical doctor always makes the final decision for diagnostic, therapeutic or prognostic use of AI | 3 (50·0) | 3.04 (1·40) | 3·0 | 1·0 | Disagree |
| 161. The introduction of AI-based diagnostic modalities will require regulatory supervision, both related to the quality of the rendered diagnosis and the ultimate destination of the diagnostic information | 7 (87·5) | 6.83 (0·48) | 7·0 | 0·0 | Very strongly agree |
| 162. Regulatory approval of AI tools used for definitive (primary) diagnosis will be very strict, but AI used for advisory purposes (secondary) will also have to meet strict regulatory conditions | 6 (70·8) | 6.04 (0·55) | 6·0 | 0·0 | Strongly agree |
| 163. CLIA regulations and clarification surrounding the use of laboratory data within pathology and laboratory processes versus outside of the laboratory will be reviewed and updated | 6 (54·2) | 5.63 (0·97) | 6·0 | 1·0 | Strongly agree |
| 164. Governments will actively promote innovation in the areas of AI and medicine, fostering the advancement of AI in pathology | 6 (58·3) | 5.88 (0·74) | 6·0 | 0·5 | Strongly agree |
| 165. Legal disputes will often arise regarding who should assume liability (pathologist, institution, developer, commercial vendor...) for diagnostic errors induced by AI | 6 (41·7) | 5.67 (1·05) | 6·0 | 1·0 | Strongly agree |
| **Section 9: Ethical aspects** | | | | | |
| *By 2030, regarding the integration of AI in pathology...* | | | | | |
| 166. As long as AI is used as a supportive method, ethical issues will be minor. However, when AI takes over tasks from the pathologist, i.e., making a diagnosis without human oversight, it will face major ethical challenges. | 7 (75·0) | 6.58 (0·93) | 7·0 | 0·5 | Very strongly agree |
|  | **Mode (%)** | **Mean (SD)** | **Median** | **IQR** | **Result** |
| 167. Other healthcare professionals will start using AI tools to diagnose cases without the aid of a pathologist | 4 , 5 (29·2) | 4.21 (1·35) | 4·0 | 1·5 | No consensus |
| 168. Pathologists will occasionally make diagnoses against their own judgment because of AI software recommendations | 5 (41·7) | 4.96 (1·00) | 5·0 | 1·5 | No consensus |
| 169. Due to the "black box" nature of many AI tools, pathologists will often make diagnoses without enough clinical explainability | 4 (37·5) | 4.38 (1·13) | 4·0 | 1·0 | Neither agree nor disagree |
| 170. Due to ethical concerns, algorithms will be prevented from making decisions in the pathology setting without additional manual review by a pathologist | 5 (45·8) | 4.92 (1·10) | 5·0 | 1·5 | No consensus |
| 171. Hurried pathologists will often take "shortcuts" by accepting AI interpretations without sufficient verification | 5 (45·8) | 5.08 (1·02) | 5·0 | 1·0 | Agree |
| 172. It will be impossible to ensure that pathologists take full responsibility for double-checking and confirming AI-rendered diagnoses | 3 (25·0) | 4.21 (1·50) | 4·0 | 2·0 | No consensus |
| 173. Pathologists will still be legally responsible for diagnoses made with the help of AI | 7 (62·5) | 6.25 (1·39) | 7·0 | 1·0 | Very strongly agree |
| 174. Potentially-biased algorithms due to lack of demographic diversity in training datasets will lead to diagnostic errors | 5 (62·5) | 5.13 (0·95) | 5·0 | 0·5 | Agree |
| 175. Limits will be imposed to unintended discoveries enabled by AI | 4 (50·0) | 4.13 (1·12) | 4·0 | 1·0 | Neither agree nor disagree |
| 176. Stricter limits will be imposed on commercialization of data | 5 (45·8) | 4.79 (1·02) | 5·0 | 1·0 | Agree |
| 177. Lack of informed patient consent when using their data will be a common practice | 5 (41·7) | 4.50 (1·10) | 5·0 | 1·0 | Agree |
| 178. Data inferences that may impact on patient anonymity will lead to ethical issues | 5 (50·0) | 5.17 (0·87) | 5·0 | 1·0 | Agree |
| 179. AI will lead to a de-skilling of pathologists, who will potentially suffer from too great a reliance on AI | 5 (33·3) | 4.54 (1·25) | 5·0 | 1·5 | No consensus |
| 180. AI and technology will be included in the educational curricula of medical students, pathologists, and analysts to help them deal with this rapidly evolving method of support and its ethical implications | 6 (62·5) | 5.88 (0·80) | 6·0 | 0·0 | Strongly agree |

For the Mode, (%) designates the percentage of panellists who selected that score. SD, standard deviation; IQR, interquartile range; AI, artificial intelligence; IT, information technology; SOP, standard operating procedure; EHR, electronic health record; IHC, immunohistochemistry; IF, immunofluorescence; QA/QC, quality assurance/quality control; AFB, acid-fast *Bacillus; H. pylori, Helicobacter pylori;* ER, estrogen receptor; PgR, progesterone receptor; PD-L1, programmed cell death ligand 1; H&E, hematoxylin and eosin; STAT6, signal transducer and activator of transcription 6; CLIA, Clinical Laboratory Improvement Amendments.
