# Supplementary material for: Computational pathology in 2030: a Delphi study forecasting the role of AI in pathology within the next decade
Source: eBioMedicine. 2023 Jan 4;88:104427. doi: 10.1016/j.ebiom.2022.104427 (PMC9823157; doi:10.1016/j.ebiom.2022.104427)
Supplement: Copy of Delphi Round 2 [file mmc2.pdf]

Delphi: Computational Pathology 2030. Round 2

Thank you for participating in Round 2 of this Delphi study.

The expert answers to Round 1 have been transformed into a series of statements. In this round, you will be asked to rate these statements according to different Likert scales.

Please answer considering only AI input, not digital pathology in a broad sense. Also, please answer according to what you believe will happen by 2030, instead of what you would like to happen.

All of your responses will remain anonymous to the rest of the panel experts.

* Required

Section 1: Impact of AI on Pathology KPIs

Please rate your AGREEMENT with the following statements according to this Likert scale:

1 Very strongly disagree
2 Strongly disagree
3 Disagree
4 Neither agree nor disagree
5 Agree
6 Strongly agree
7 Very strongly agree

By 2030, due to the integration of AI in the pathology setting...

1. 1. Time required per case will decrease *

Mark only one oval.

	Very strongly disagree
1	⬭
2	⬭
3	⬭
4	⬭
5	⬭
6	⬭
7	⬭
	Very strongly agree

2. 2. Cost per case will decrease *

Mark only one oval.

Very strongly disagree	
1	⬭
2	⬭
3	⬭
4	⬭
5	⬭
6	⬭
7	⬭
Very strongly agree	

3. 3. Standardization of pre-analytical processes (staining and slicing techniques) will increase *

Mark only one oval.

	Very strongly disagree
1	⬭
2	⬭
3	⬭
4	⬭
5	⬭
6	⬭
7	⬭
	Very strongly agree

4. 4. The number of unnecessary IHC stains performed will decrease *

Mark only one oval.

Very strongly disagree

1 ⬭

2 ⬭

3 ⬭

4 ⬭

5 ⬭

6 ⬭

7 ⬭

Very strongly agree

5. 5. Pathologists will look at a substantially lower number of cases, since cases will be filtered (such as negative biopsies) *

Mark only one oval.

	Very strongly disagree
1	⬭
2	⬭
3	⬭
4	⬭
5	⬭
6	⬭
7	⬭
	Very strongly agree

6. 6. Diagnostic accuracy will increase *

Mark only one oval.

Very strongly disagree

1 ⬭

2 ⬭

3 ⬭

4 ⬭

5 ⬭

6 ⬭

7 ⬭

Very strongly agree

7. 7. Diagnosis and grading of tumors will be more standardized, bringing more objectivity to the diagnosis of certain entities that are currently subject to high interobserver variability *

Mark only one oval.

	Very strongly disagree
1	◯
2	◯
3	◯
4	◯
5	◯
6	◯
7	◯
	Very strongly agree

8. 8. Detection of rare events (small metastases, small tumor foci) will increase *

Mark only one oval.

	Very strongly disagree
1	⬭
2	⬭
3	⬭
4	⬭
5	⬭
6	⬭
7	⬭
	Very strongly agree

9. 9. Analyses will be more quantitative *

Mark only one oval.

	Very strongly disagree
1	⬭
2	⬭
3	⬭
4	⬭
5	⬭
6	⬭
7	⬭
	Very strongly agree

10. 10. Completeness of reports will increase *

Mark only one oval.

	Very strongly disagree
1	⬭
2	⬭
3	⬭
4	⬭
5	⬭
6	⬭
7	⬭
	Very strongly agree

11. 11. Complexity of reports will increase *

Mark only one oval.

Very strongly disagree

1 ⬭

2 ⬭

3 ⬭

4 ⬭

5 ⬭

6 ⬭

7 ⬭

Very strongly agree

12. 12. Quality of reports will increase *

Mark only one oval.

	Very strongly disagree
1	⬭
2	⬭
3	⬭
4	⬭
5	⬭
6	⬭
7	⬭
	Very strongly agree

13. 13. The number of second-opinion consultations will decrease *

Mark only one oval.

	Very strongly disagree
1	◯
2	◯
3	◯
4	◯
5	◯
6	◯
7	◯
	Very strongly agree

14. 14. Satisfaction of referring physicians will increase *

Mark only one oval.

Very strongly disagree	
1	◯
2	◯
3	◯
4	◯
5	◯
6	◯
7	◯
Very strongly agree	

15. 15. Patient satisfaction will increase *

Mark only one oval.

Very strongly disagree

1 ⬭
2 ⬭
3 ⬭
4 ⬭
5 ⬭
6 ⬭
7 ⬭

Very strongly agree

Section 2: Impact of AI on the Pathology workforce

Please estimate how the integration of AI in the Pathology setting will impact the workforce, by rating the following statements according to this Likert scale:

1 Dramatically decrease (from -50% to -100%)
2 Greatly decrease (from -20% to -50%)
3 Somewhat decrease (from -5% to -20%)
4 Remain the same (from -5% to +5%)
5 Somewhat increase (from +5% to +20%)
6 Greatly increase (from +20% to +50%)
7 Dramatically increase (from +50% to +100%)

By 2030, due to the integration of AI in the pathology setting...

16. 16. The number of jobs for pathologists will *

Mark only one oval.

	Dramatically decrease
1	⬭
2	⬭
3	⬭
4	⬭
5	⬭
6	⬭
7	⬭
	Dramatically increase

17. 17. The number of jobs for pathology technicians will *

Mark only one oval.

Dramatically decrease	
1	⬭
2	⬭
3	⬭
4	⬭
5	⬭
6	⬭
7	⬭
Dramatically increase	

18. 18. The number of jobs for IT staff will *

Mark only one oval.

	Dramatically decrease
1	⬭
2	⬭
3	⬭
4	⬭
5	⬭
6	⬭
7	⬭
	Dramatically increase

19. 19. The number of jobs in administrative positions will *

Mark only one oval.

	Dramatically decrease
1	⬭
2	⬭
3	⬭
4	⬭
5	⬭
6	⬭
7	⬭
	Dramatically increase

20. 20. The ratio of general pathologists will *

Mark only one oval.

	Dramatically decrease
1	◯
2	◯
3	◯
4	◯
5	◯
6	◯
7	◯
	Dramatically increase

21. 21. The ratio of subspecialized pathologists will *

Mark only one oval.

	Dramatically decrease
1	⬭
2	⬭
3	⬭
4	⬭
5	⬭
6	⬭
7	⬭
	Dramatically increase

22. 22. The number of specialized "computational" pathologists will *

Mark only one oval.

	Dramatically decrease
1	⬭
2	⬭
3	⬭
4	⬭
5	⬭
6	⬭
7	⬭
	Dramatically increase

23. 23. The number of overall jobs in Pathology will *

Mark only one oval.

	Dramatically decrease
1	◯
2	◯
3	◯
4	◯
5	◯
6	◯
7	◯
	Dramatically increase

24. 24. Beyond 2030 (by 2040), the number of jobs for pathologists will *

Mark only one oval.

	Dramatically decrease
1	◯
2	◯
3	◯
4	◯
5	◯
6	◯
7	◯
	Dramatically increase

25. 25. Beyond 2030 (by 2040), the overall number of jobs in Pathology will *

Mark only one oval.

	Dramatically decrease
1	( )
2	( )
3	( )
4	( )
5	( )
6	( )
7	( )
	Dramatically increase

Now, please rate your AGREEMENT with the following statements according to this Likert scale:

1 Very strongly disagree
2 Strongly disagree
3 Disagree
4 Neither agree nor disagree
5 Agree
6 Strongly agree
7 Very strongly agree

By 2030, due to the integration of AI in the pathology setting...

26. 26. Practices with retiring pathologists may choose not to replace them *

Mark only one oval.

	Very strongly disagree
1	⬭
2	⬭
3	⬭
4	⬭
5	⬭
6	⬭
7	⬭
	Very strongly agree

27. 27. AI will pose an increased incentive to retire, for those pathologists who are unwilling to apply AI to their daily job *

Mark only one oval.

	Very strongly disagree
1	◯
2	◯
3	◯
4	◯
5	◯
6	◯
7	◯
	Very strongly agree

28. 28. Pathology will attract more and better talent into the specialty, because of an increased interest due to AI developments in the field *

Mark only one oval.

	Very strongly disagree
1	⬭
2	⬭
3	⬭
4	⬭
5	⬭
6	⬭
7	⬭
	Very strongly agree

Section 3: Tasks of pathologists

Please estimate the degree of involvement of pathologists in these tasks by 2030, by rating the following statements according to this Likert scale:

1 Not involved at all
2 Rarely involved
3 Somewhat involved
4 Sometimes involved
5 Often involved
6 Routinely involved
7 Involved daily

By 2030, due to the integration of AI in the pathology setting, the degree of involvement of pathologists in these tasks will be…

29. 29. Digital pathologic diagnosis without the use of physical glass slides *

Mark only one oval.

Not involved at all

1

2

3

4

5

6

7

Involved daily

30. 30. Interpretation of computationally derived measurements and evaluations *

Mark only one oval.

Not involved at all

1

2

3

4

5

6

7

Involved daily

31. 31. Collaboration with EHR teams regarding the use of laboratory data for a wide range of clinical decision support tools *

Mark only one oval.

	Not involved at all
1	⬭
2	⬭
3	⬭
4	⬭
5	⬭
6	⬭
7	⬭
	Involved daily

32. 32. Direct patient care activities *

Mark only one oval.

	Not involved at all
1	◯
2	◯
3	◯
4	◯
5	◯
6	◯
7	◯
	Involved daily

33. 33. Decision-making related to patient treatment *

Mark only one oval.

	Not involved at all
1	⬭
2	⬭
3	⬭
4	⬭
5	⬭
6	⬭
7	⬭
	Involved daily

34. 34. Patient identification for clinical trials *

Mark only one oval.

Not involved at all

1 ⬭
2 ⬭
3 ⬭
4 ⬭
5 ⬭
6 ⬭
7 ⬭

Involved daily

35. 35. Evaluating different kinds of AI software and deciding whether these are appropriate for their workflow *

Mark only one oval.

	Not involved at all
1	⬭
2	⬭
3	⬭
4	⬭
5	⬭
6	⬭
7	⬭
	Involved daily

36. 36. Validation and QA/QC of AI solutions *

Mark only one oval.

Not involved at all

1 ◯

2 ◯

3 ◯

4 ◯

5 ◯

6 ◯

7 ◯

Involved daily

37. 37. Validation and QA/QC of AI-rendered diagnoses *

Mark only one oval.

	Not involved at all
1	⬭
2	⬭
3	⬭
4	⬭
5	⬭
6	⬭
7	⬭
	Involved daily

38. 38. Defining new categories of patients, based on new data made available through AI *

Mark only one oval.

	Not involved at all
1	◯
2	◯
3	◯
4	◯
5	◯
6	◯
7	◯
	Involved daily

39. 39. Design of AI solutions *

Mark only one oval.

	Not involved at all
1	◯
2	◯
3	◯
4	◯
5	◯
6	◯
7	◯
	Involved daily

40. 40. Development of AI solutions, including annotation of image data and algorithm training *

Mark only one oval.

	Not involved at all
1	⬭
2	⬭
3	⬭
4	⬭
5	⬭
6	⬭
7	⬭
	Involved daily

41. 41. Mass spectrometry analyses *

Mark only one oval.

	Not involved at all
1	⬭
2	⬭
3	⬭
4	⬭
5	⬭
6	⬭
7	⬭
	Involved daily

42. 42. Molecular pathology, including NGS analyses *

Mark only one oval.

	Not involved at all
1	⬭
2	⬭
3	⬭
4	⬭
5	⬭
6	⬭
7	⬭
	Involved daily

43. 43. In situ genetic analyses using multiplex technology *

Mark only one oval.

Not involved at all

1 ⬭
2 ⬭
3 ⬭
4 ⬭
5 ⬭
6 ⬭
7 ⬭

Involved daily

Now, please rate your AGREEMENT with the following statements according to this Likert scale:

1 Very strongly disagree
2 Strongly disagree
3 Disagree
4 Neither agree nor disagree
5 Agree
6 Strongly agree
7 Very strongly agree

44. 44. Pathologists will be more involved in diagnostic tumor boards *

Mark only one oval.

	Very strongly disagree
1	⬭
2	⬭
3	⬭
4	⬭
5	⬭
6	⬭
7	⬭
	Very strongly agree

45. 45 Pathologists will be more involved in multidisciplinary conferences *

Mark only one oval.

Very strongly disagree	
1	⬭
2	⬭
3	⬭
4	⬭
5	⬭
6	⬭
7	⬭

Very strongly agree

46. 46. Pathologists will be more involved in research activities *

Mark only one oval.

	Very strongly disagree
1	⬭
2	⬭
3	⬭
4	⬭
5	⬭
6	⬭
7	⬭
	Very strongly agree

47. 47. Pathologists will be spending more time in the study of rare lesions *

Mark only one oval.

Very strongly disagree

1 ○
2 ○
3 ○
4 ○
5 ○
6 ○
7 ○

Very strongly agree

Section 4: Tasks of pathology technicians

Please estimate the degree of involvement of pathology technicians in these tasks by 2030, by rating the following statements according to this Likert scale:

1 Not involved at all
2 Rarely involved
3 Somewhat involved
4 Sometimes involved
5 Often involved
6 Routinely involved
7 Involved daily

By 2030, due to the integration of AI in the pathology setting, the degree of involvement of pathology technicians in these tasks will be…

48. 48. Operation of digital slide scanners, digitization and image management *

Mark only one oval.

Not involved at all

1 ( )

2 ( )

3 ( )

4 ( )

5 ( )

6 ( )

7 ( )

Involved daily

49. 49. QA/QC of digitized images *

Mark only one oval.

	Not involved at all
1	⬭
2	⬭
3	⬭
4	⬭
5	⬭
6	⬭
7	⬭
	Involved daily

50. 50. Digital Pathology support for pathologists and other users, such as device calibration *

Mark only one oval.

Not involved at all

1 ⬭

2 ⬭

3 ⬭

4 ⬭

5 ⬭

6 ⬭

7 ⬭

Involved daily

51. 51. Assessing histology consistency, i.e. re-addressing SOPs to make slides and corresponding images more suitable for AI (more consistent tissue and staining quality) *

Mark only one oval.

	Not involved at all
1	⬭
2	⬭
3	⬭
4	⬭
5	⬭
6	⬭
7	⬭
	Involved daily

52. 52. Sample grossing guided by AI-derived image analysis *

Mark only one oval.

	Not involved at all
1	⬭
2	⬭
3	⬭
4	⬭
5	⬭
6	⬭
7	⬭
	Involved daily

53. 53. Diagnosis of cervical cancer aided by AI *

Mark only one oval.

Not involved at all

1 ○
2 ○
3 ○
4 ○
5 ○
6 ○
7 ○

Involved daily

54. 54. Diagnosis of other diseases, aided by AI *

Mark only one oval.

Not involved at all

1

2

3

4

5

6

7

Involved daily

55. 55. Counting of quantitative markers, such as Ki-67 *

Mark only one oval.

	Not involved at all
1	⬭
2	⬭
3	⬭
4	⬭
5	⬭
6	⬭
7	⬭
	Involved daily

56. 56. Validation and QA/QC of AI-rendered diagnoses *

Mark only one oval.

	Not involved at all
1	⬭
2	⬭
3	⬭
4	⬭
5	⬭
6	⬭
7	⬭
	Involved daily

57. 57. Development of AI solutions, including annotation of image data and algorithm training *

Mark only one oval.

	Not involved at all
1	◯
2	◯
3	◯
4	◯
5	◯
6	◯
7	◯
	Involved daily

58. 58. Preparation of reports *

Mark only one oval.

	Not involved at all
1	◯
2	◯
3	◯
4	◯
5	◯
6	◯
7	◯
	Involved daily

Section 5: AI applications in Pathology

Please estimate the PROBABILITY of these applications of AI being used routinely in pathology labs by 2030, by rating the following statements according to this Likert scale:

1 Impossible (0)
2 Very unlikely (0 - 0.2)
3 Unlikely (0.2 - 0.4)
4 Even chance/neutral (0.4 - 0.6)
5 Likely (0.6 - 0.8)
6 Very likely (0.8 - 1)
7 Certain (1)

By 2030, the probability of these AI tools being used routinely in pathology labs is:

59. 59. AI-assisted laboratory workflow management, including workload assignments * to pathologists, residents, and technicians

Mark only one oval.

	Impossible
1	⬭
2	⬭
3	⬭
4	⬭
5	⬭
6	⬭
7	⬭
	Certain

60. 60. Automatic QA/QC of macroscopic images and grossing *

Mark only one oval.

	Impossible
1	◯
2	◯
3	◯
4	◯
5	◯
6	◯
7	◯
	Certain

61. 61. Automated ordering of IHC for specific applications / assisting with selection of * immunohistochemical stains needed

Mark only one oval.

	Impossible
1	◯
2	◯
3	◯
4	◯
5	◯
6	◯
7	◯
	Certain

62. 62. Automated QA/QC of IHC positive and negative controls *

Mark only one oval.

	Impossible
1	⬭
2	⬭
3	⬭
4	⬭
5	⬭
6	⬭
7	⬭
	Certain

63. 63. Pre-selection of potentially cancer-positive samples for pathologist's review, while the bulk of clearly negative samples can be automatically processed *

Mark only one oval.

	Impossible
1	◯
2	◯
3	◯
4	◯
5	◯
6	◯
7	◯
	Certain

64. 64. Triaging of cases to the most appropriate pathologist at the earliest possible time *

Mark only one oval.

	Impossible
1	⬭
2	⬭
3	⬭
4	⬭
5	⬭
6	⬭
7	⬭
	Certain

65. 65. Generation of H&E-style WSI directly from fresh tissue (bypassing the need for frozen section slide creation) *

Mark only one oval.

Impossible

1 ◯

2 ◯

3 ◯

4 ◯

5 ◯

6 ◯

7 ◯

Certain

66. 66. Up-front case classification associated with IHC selection and followed by triaging to the specialist *

Mark only one oval.

	Impossible
1	◯
2	◯
3	◯
4	◯
5	◯
6	◯
7	◯
	Certain

67. 67. Computer handling of routine specimens, thus freeing up time for pathologists to handle difficult cases *

Mark only one oval.

	Impossible
1	◯
2	◯
3	◯
4	◯
5	◯
6	◯
7	◯
	Certain

68. 68. Proposing specific IHC or other molecular methods to solve a specific diagnostic problem *

Mark only one oval.

	Impossible
1	⬭
2	⬭
3	⬭
4	⬭
5	⬭
6	⬭
7	⬭
	Certain

69. 69. Prioritization of cases (such as cases with neoplasia and infectious organisms in immunosuppressed patients) *

Mark only one oval.

	Impossible
1	◯
2	◯
3	◯
4	◯
5	◯
6	◯
7	◯
	Certain

70. 70. Generation of gross descriptions of pathology specimens *

Mark only one oval.

Impossible

1

2

3

4

5

6

7

Certain

71. 71. Identification of mislabeled specimens (e.g. a prostate biopsy case containing tissue from a different organ) *

Mark only one oval.

	Impossible
1	◯
2	◯
3	◯
4	◯
5	◯
6	◯
7	◯
	Certain

72. 72. Automated routing of cases for workup by robotics *

Mark only one oval.

	Impossible
1	
2	
3	
4	
5	
6	
7	
	Certain

73. 73. Quality control of whole-slide images (scanning process), and detection of poor-quality slides (tissue folds, poor staining). *

Mark only one oval.

Impossible

1 ◯

2 ◯

3 ◯

4 ◯

5 ◯

6 ◯

7 ◯

Certain

74. 74. Quality improvement of whole-slide images *

Mark only one oval.

	Impossible
1	⬭
2	⬭
3	⬭
4	⬭
5	⬭
6	⬭
7	⬭
	Certain

75. 75. Tools that bring up similar cases with their respective diagnoses for feature comparison *

Mark only one oval.

	Impossible
1	⬭
2	⬭
3	⬭
4	⬭
5	⬭
6	⬭
7	⬭
	Certain

76. 76. Pre-selecting regions of interest suspicious for cancer for pathologists to view *

Mark only one oval.

Impossible

1 ○

2 ○

3 ○

4 ○

5 ○

6 ○

7 ○

Certain

77. 77. Identification of hotspot areas *

Mark only one oval.

	Impossible
1	
2	
3	
4	
5	
6	
7	
	Certain

78. 78. Identification of micrometastases *

Mark only one oval.

	Impossible
1	⬭
2	⬭
3	⬭
4	⬭
5	⬭
6	⬭
7	⬭
	Certain

79. 79. Detection of lymph node metastases *

Mark only one oval.

	Impossible
1	◯
2	◯
3	◯
4	◯
5	◯
6	◯
7	◯
	Certain

80. 80. Detection of signet ring-cell cancer *

Mark only one oval.

	Impossible
1	⬭
2	⬭
3	⬭
4	⬭
5	⬭
6	⬭
7	⬭
	Certain

81. 81. Detection of microorganisms (AFB, *H. pylori*) *

Mark only one oval.

	Impossible
1	⬭
2	⬭
3	⬭
4	⬭
5	⬭
6	⬭
7	⬭
	Certain

82. 82. Assisting with tumor grading *

Mark only one oval.

	Impossible
1	◯
2	◯
3	◯
4	◯
5	◯
6	◯
7	◯
	Certain

83. 83. Identification of molecular biomarker status directly from H&E WSI: automated mutation detection in cancer, such as non-small cell lung cancer *

Mark only one oval.

	Impossible
1	⬭
2	⬭
3	⬭
4	⬭
5	⬭
6	⬭
7	⬭
	Certain

84. 84. Identification of molecular biomarker status directly from H&E WSI: prediction of clinical outcome and response to treatment, e.g. in colorectal cancer *

Mark only one oval.

	Impossible
1	⬭
2	⬭
3	⬭
4	⬭
5	⬭
6	⬭
7	⬭
	Certain

85. 85. Quantification of IHC or IF stains, such as Ki-67, ER, PgR, PD-L1 *

Mark only one oval.

	Impossible
1	⬭
2	⬭
3	⬭
4	⬭
5	⬭
6	⬭
7	⬭
	Certain

86. 86. Quantification of number of mitoses in H&E stained images *

Mark only one oval.

	Impossible
1	⬭
2	⬭
3	⬭
4	⬭
5	⬭
6	⬭
7	⬭
	Certain

87. 87. Counting lymphocytes *

Mark only one oval.

	Impossible
1	○
2	○
3	○
4	○
5	○
6	○
7	○
	Certain

88. 88. Quantification of eosinophils in eosinophilic esophagitis *

Mark only one oval.

Impossible

1 ⬭

2 ⬭

3 ⬭

4 ⬭

5 ⬭

6 ⬭

7 ⬭

Certain

89. 89. Quantitation of features (e.g., fibrosis in various organs, liver steatosis, etc.) *

Mark only one oval.

	Impossible
1	⬭
2	⬭
3	⬭
4	⬭
5	⬭
6	⬭
7	⬭
	Certain

90. 90. Marking of perineural invasion, lymphovascular invasion *

Mark only one oval.

	Impossible
1	⬭
2	⬭
3	⬭
4	⬭
5	⬭
6	⬭
7	⬭
	Certain

91. 91. Allowing for computational staining instead of multiplexing *

Mark only one oval.

	Impossible
1	⬭
2	⬭
3	⬭
4	⬭
5	⬭
6	⬭
7	⬭
	Certain

92. 92. Providing a set of differential diagnoses on difficult cases *

Mark only one oval.

	Impossible
1	⬭
2	⬭
3	⬭
4	⬭
5	⬭
6	⬭
7	⬭
	Certain

93. 93. Proposing specific additional tests for solving a diagnostic problem (e.g. AI algorithm suggesting STAT6 immunostaining on a spindle cell neoplasm of the pleura) *

Mark only one oval.

	Impossible
1	◯
2	◯
3	◯
4	◯
5	◯
6	◯
7	◯
	Certain

94. 94. Automated measurements (e.g., of tumor areas) *

Mark only one oval.

	Impossible
1	◯
2	◯
3	◯
4	◯
5	◯
6	◯
7	◯
	Certain

95. 95. Ensuring all diagnostically relevant areas on the slide are viewed prior to report finalization *

Mark only one oval.

	Impossible
1	⬭
2	⬭
3	⬭
4	⬭
5	⬭
6	⬭
7	⬭
	Certain

96. 96. Import of contextually-related data on a case for quick review by the pathologist during diagnostic slide review *

Mark only one oval.

	Impossible
1	◯
2	◯
3	◯
4	◯
5	◯
6	◯
7	◯
	Certain

97. 97. Mandatory second reads when the pathologist diagnosis does not match the potential AI diagnosis (within a predefined range/percentage; e.g., if the AI tool detects potential tumor on a biopsy but the pathologist reads the biopsy as no evidence of tumor) *

Mark only one oval.

Impossible

1 ⬭

2 ⬭

3 ⬭

4 ⬭

5 ⬭

6 ⬭

7 ⬭

Certain

98. 98. Standardization of pathology reports *

Mark only one oval.

	Impossible
1	⬭
2	⬭
3	⬭
4	⬭
5	⬭
6	⬭
7	⬭
	Certain

99. 99. Pre-populating relevant report details from the medical record/gross description *

Mark only one oval.

Impossible
1
2
3
4
5
6
7
Certain

100. 100. Selection of the appropriate synoptic report based on prior pathology findings, including the current case gross report *

Mark only one oval.

	Impossible
1	⬭
2	⬭
3	⬭
4	⬭
5	⬭
6	⬭
7	⬭
	Certain

101. 101. Pre-populating reports based on AI interpretation of images *

Mark only one oval.

	Impossible
1	◯
2	◯
3	◯
4	◯
5	◯
6	◯
7	◯
	Certain

102. 102. Finding the source of contaminants *

Mark only one oval.

	Impossible
1	⬭
2	⬭
3	⬭
4	⬭
5	⬭
6	⬭
7	⬭
	Certain

Section 6: Role of AI in integrated diagnostics

Please estimate the PROBABILITY of these applications of integrated diagnostics being used routinely by 2030, by rating the following statements according to this Likert scale:

1 Impossible (0)
2 Very unlikely (0 - 0.2)
3 Unlikely (0.2 - 0.4)
4 Even chance/neutral (0.4 - 0.6)
5 Likely (0.6 - 0.8)
6 Very likely (0.8 - 1)
7 Certain (1)

By 2030, the probability of these integrated diagnostic applications being used routinely is:

103. 103. Comparison of tumor extent in slides and radiological images *

Mark only one oval.

Impossible

1 ⬭

2 ⬭

3 ⬭

4 ⬭

5 ⬭

6 ⬭

7 ⬭

Certain

104. 104. Identification of histologic regions to be sampled for genomic testing *

Mark only one oval.

	Impossible
1	◯
2	◯
3	◯
4	◯
5	◯
6	◯
7	◯
	Certain

105. 105. Prompting of further genetic testing based on AI analysis of histology *

Mark only one oval.

	Impossible
1	⬭
2	⬭
3	⬭
4	⬭
5	⬭
6	⬭
7	⬭
	Certain

106. 106. Assessment of staging based on combined pathology/genetic data *

Mark only one oval.

	Impossible
1	
2	
3	
4	
5	
6	
7	
	Certain

107. 107. Correlation of morphological and genomic information in order to interpret genetic aberrations found *

Mark only one oval.

	Impossible
1	⬭
2	⬭
3	⬭
4	⬭
5	⬭
6	⬭
7	⬭
	Certain

108. 108. Extraction of molecular data from radiological or histological images *

Mark only one oval.

	Impossible
1	⬭
2	⬭
3	⬭
4	⬭
5	⬭
6	⬭
7	⬭
	Certain

109. 109. Prediction of biomarker status and clinical outcomes for personalized medicine, based on integrated diagnostics *

Mark only one oval.

	Impossible
1	◯
2	◯
3	◯
4	◯
5	◯
6	◯
7	◯
	Certain

110. 110. Identifying discrepancies between radiologic and pathologic diagnoses *

Mark only one oval.

	Impossible
1	
2	
3	
4	
5	
6	
7	
	Certain

111. 111. Algorithms for interpretation of tumor treatment response in radiology, based on pathology data *

Mark only one oval.

	Impossible
1	◯
2	◯
3	◯
4	◯
5	◯
6	◯
7	◯
	Certain

112. 112. Algorithms that transfer pathology diagnostic information to radiologic images: Based on the pathology diagnosis, a heatmap is shown on radiology images, displaying tumor magnitudes *

Mark only one oval.

Impossible

1 ⬭

2 ⬭

3 ⬭

4 ⬭

5 ⬭

6 ⬭

7 ⬭

Certain

113. 113. Combination of all data (macro, micro, radiology, genomic) on the same screen *

Mark only one oval.

	Impossible
1	⬭
2	⬭
3	⬭
4	⬭
5	⬭
6	⬭
7	⬭
	Certain

114. 114. Diagnoses based on combination of genomics, radiomics and multiparameter IHC along with blood counts and serum analysis *

Mark only one oval.

	Impossible
1	◯
2	◯
3	◯
4	◯
5	◯
6	◯
7	◯
	Certain

115. 115. Displaying a list of the most probable diagnoses for a particular case, according to the combination of different diagnostic techniques *

Mark only one oval.

	Impossible
1	◯
2	◯
3	◯
4	◯
5	◯
6	◯
7	◯
	Certain

116. 116. Reduction of healthcare costs by avoiding redundancy of testing *

Mark only one oval.

	Impossible
1	⬭
2	⬭
3	⬭
4	⬭
5	⬭
6	⬭
7	⬭
	Certain

117. 117. Clinical use of prediction models resulting from mutlivariable analysis of data from different modalities (pathology, radiology, genetics) *

Mark only one oval.

	Impossible
1	◯
2	◯
3	◯
4	◯
5	◯
6	◯
7	◯
	Certain

118. 118. Selection of patients with prostate cancer for active surveillance versus radiotherapy/surgery, based on integration of pathology and radiology data *

Mark only one oval.

	Impossible
1	⬭
2	⬭
3	⬭
4	⬭
5	⬭
6	⬭
7	⬭
	Certain

119. 119. Creation of new categories of patients by integrating all "big data" from pathology, clinical lab, radiology and genomics *

Mark only one oval.

	Impossible
1	◯
2	◯
3	◯
4	◯
5	◯
6	◯
7	◯
	Certain

120. 120. Building risk stratification (prognostic) roadmaps for individual patients based on input from histology, radiology, and genomics *

Mark only one oval.

	Impossible
1	⬭
2	⬭
3	⬭
4	⬭
5	⬭
6	⬭
7	⬭
	Certain

121. 121. Use of integrated reports for select conditions, e.g., prostate cancer *

Mark only one oval.

Impossible

1 ⬭

2 ⬭

3 ⬭

4 ⬭

5 ⬭

6 ⬭

7 ⬭

Certain

Section 7: Pathology tasks fully automated by AI in 2030

Please estimate the PROBABILITY that these tasks will become FULLY DELEGATED to AI and thus done in a FULLY AUTOMATED WAY in pathology labs by 2030, by rating the following statements according to this Likert scale:

1 Impossible (0)
2 Very unlikely (0 - 0.2)
3 Unlikely (0.2 - 0.4)
4 Even chance/neutral (0.4 - 0.6)
5 Likely (0.6 - 0.8)
6 Very likely (0.8 - 1)
7 Certain (1)

By 2030, the probability of these tasks being fully delegated to AI in pathology labs is...

122. 122. Screening of tissues with a cancer diagnosis to select regions for tissue coring or macroscopic dissection *

Mark only one oval.

	Impossible
1	⬭
2	⬭
3	⬭
4	⬭
5	⬭
6	⬭
7	⬭
	Certain

123. 123. Selection of which IHC to be performed *

Mark only one oval.

	Impossible
1	◯
2	◯
3	◯
4	◯
5	◯
6	◯
7	◯
	Certain

124. 124. Verification of positive and negative controls for IHC *

Mark only one oval.

	Impossible
1	⬭
2	⬭
3	⬭
4	⬭
5	⬭
6	⬭
7	⬭
	Certain

125. 125. Prioritization of cases *

Mark only one oval.

	Impossible
1	⬭
2	⬭
3	⬭
4	⬭
5	⬭
6	⬭
7	⬭
	Certain

126. 126. Triaging of cases to appropriate pathologists *

Mark only one oval.

	Impossible
1	◯
2	◯
3	◯
4	◯
5	◯
6	◯
7	◯
	Certain

127. 127. Contextual data lookup on patients from the EHR relevant to the pathology case being reviewed *

Mark only one oval.

	Impossible
1	◯
2	◯
3	◯
4	◯
5	◯
6	◯
7	◯
	Certain

128. 128. Slide QC (e.g., detection of tissue folds and tears, stain quality evaluation, etc.) *

Mark only one oval.

	Impossible
1	◯
2	◯
3	◯
4	◯
5	◯
6	◯
7	◯
	Certain

129. 129. Screening of microorganisms, such as AFB and *H. pylori* *

Mark only one oval.

Impossible

1 ⬭

2 ⬭

3 ⬭

4 ⬭

5 ⬭

6 ⬭

7 ⬭

Certain

130. 130. Screening of colorectal polyps *

Mark only one oval.

	Impossible
1	⬭
2	⬭
3	⬭
4	⬭
5	⬭
6	⬭
7	⬭
	Certain

131. 131. Cervical cytology screening *

Mark only one oval.

	Impossible
1	◯
2	◯
3	◯
4	◯
5	◯
6	◯
7	◯
	Certain

132. 132. Screening lymph nodes for metastases *

Mark only one oval.

	Impossible
1	
2	
3	
4	
5	
6	
7	
	Certain

133. 133. Detection of tumors in H&E-stained WSI *

Mark only one oval.

	Impossible
1	⬭
2	⬭
3	⬭
4	⬭
5	⬭
6	⬭
7	⬭
	Certain

134. 134. Slide screening for regions of interest *

Mark only one oval.

	Impossible
1	⬭
2	⬭
3	⬭
4	⬭
5	⬭
6	⬭
7	⬭
	Certain

135. 135. Measurement tasks *

Mark only one oval.

	Impossible
1	⬭
2	⬭
3	⬭
4	⬭
5	⬭
6	⬭
7	⬭
	Certain

136. 136. Assessing tumor cellularity *

Mark only one oval.

	Impossible
1	⬭
2	⬭
3	⬭
4	⬭
5	⬭
6	⬭
7	⬭
	Certain

137. 137. Quantification of IHC or IF stains, such as Ki-67, ER, PgR, PD-L1 *

Mark only one oval.

	Impossible
1	⬭
2	⬭
3	⬭
4	⬭
5	⬭
6	⬭
7	⬭
	Certain

138. 138. Quantification of mitotic count on H&E-stained images *

Mark only one oval.

	Impossible
1	⬭
2	⬭
3	⬭
4	⬭
5	⬭
6	⬭
7	⬭
	Certain

139. 139. Bone marrow differential counts *

Mark only one oval.

	Impossible
1	⬭
2	⬭
3	⬭
4	⬭
5	⬭
6	⬭
7	⬭
	Certain

140. 140. Classification of skin lesions *

Mark only one oval.

	Impossible
1	◯
2	◯
3	◯
4	◯
5	◯
6	◯
7	◯
	Certain

141. 141. MIB-1 scoring *

Mark only one oval.

	Impossible
1	⬭
2	⬭
3	⬭
4	⬭
5	⬭
6	⬭
7	⬭
	Certain

142. 142. Grading of dysplasia *

Mark only one oval.

	Impossible
1	◯
2	◯
3	◯
4	◯
5	◯
6	◯
7	◯
	Certain

143. 143. Assessing extent of liver steatosis and fibrosis *

Mark only one oval.

Impossible

1

2

3

4

5

6

7

Certain

144. 144. Grading of prostate cancer *

Mark only one oval.

	Impossible
1	
2	
3	
4	
5	
6	
7	
	Certain

145. 145. Grading of breast cancer *

Mark only one oval.

	Impossible
1	
2	
3	
4	
5	
6	
7	
	Certain

146. 146. Grading of colorectal cancer *

Mark only one oval.

	Impossible
1	◯
2	◯
3	◯
4	◯
5	◯
6	◯
7	◯
	Certain

147. 147. Grading of lung cancer *

Mark only one oval.

	Impossible
1	⬭
2	⬭
3	⬭
4	⬭
5	⬭
6	⬭
7	⬭
	Certain

148. 148. Integration of data from multiple IHC stains *

Mark only one oval.

	Impossible
1	⬭
2	⬭
3	⬭
4	⬭
5	⬭
6	⬭
7	⬭
	Certain

149. 149. Correlation of morphologic, immunofluorescence and molecular pathology images *

Mark only one oval.

	Impossible
1	◯
2	◯
3	◯
4	◯
5	◯
6	◯
7	◯
	Certain

Section 8: Regulatory aspects

Please rate your AGREEMENT with the following statements according to this Likert scale:

1 Very strongly disagree
2 Strongly disagree
3 Disagree
4 Neither agree nor disagree
5 Agree
6 Strongly agree
7 Very strongly agree

By 2030, regarding the integration of AI in pathology ...

150. 150. A set of new guidelines will be developed, specifically addressing the integration of AI in pathology *

Mark only one oval.

	Very strongly disagree
1	⬭
2	⬭
3	⬭
4	⬭
5	⬭
6	⬭
7	⬭
	Very strongly agree

151. 151. Specific validation procedures for different types of AI tools will be defined by regulatory bodies *

Mark only one oval.

	Very strongly disagree
1	⬭
2	⬭
3	⬭
4	⬭
5	⬭
6	⬭
7	⬭
	Very strongly agree

152. 152. Regulatory pathways concerning AI tools for pathology will be simplified *

Mark only one oval.

	Very strongly disagree
1	⬭
2	⬭
3	⬭
4	⬭
5	⬭
6	⬭
7	⬭
	Very strongly agree

153. 153. Approving developers once, circumventing the need to approve every app developed by a cleared AI company, will be possible *

Mark only one oval.

	Very strongly disagree
1	⬭
2	⬭
3	⬭
4	⬭
5	⬭
6	⬭
7	⬭
	Very strongly agree

154. 154. Validation of families of algorithms with the same basic structure, rather than on an individual basis for each algorithm and application, will be possible *

Mark only one oval.

	Very strongly disagree
1	⬭
2	⬭
3	⬭
4	⬭
5	⬭
6	⬭
7	⬭
	Very strongly agree

155. 155. Approving adaptive algorithms that constantly change will be possible *

Mark only one oval.

	Very strongly disagree
1	⬭
2	⬭
3	⬭
4	⬭
5	⬭
6	⬭
7	⬭
	Very strongly agree

156. 156. Any legal and administrative barriers to use of anonymized images in education and research will be overcome *

Mark only one oval.

	Very strongly disagree
1	◯
2	◯
3	◯
4	◯
5	◯
6	◯
7	◯
	Very strongly agree

157. 157. Meeting regulatory requirements for most AI applications will be a lengthy and costly process, as it will involve large-scale prospective studies *

Mark only one oval.

	Very strongly disagree
1	⬭
2	⬭
3	⬭
4	⬭
5	⬭
6	⬭
7	⬭
	Very strongly agree

158. 158. Definition of endpoints for clinical validation studies will be a common problem *

Mark only one oval.

	Very strongly disagree
1	⬭
2	⬭
3	⬭
4	⬭
5	⬭
6	⬭
7	⬭
	Very strongly agree

159. 159. Post-marketing surveilance will pose important challenges, due to algorithm drift *

Mark only one oval.

	Very strongly disagree
1	⬭
2	⬭
3	⬭
4	⬭
5	⬭
6	⬭
7	⬭
	Very strongly agree

160. 160. Regulatory issues will not be a challenge for AI use in pathology, since the medical doctor always makes the final decision for diagnostic, therapeutic or prognostic use of AI *

Mark only one oval.

	Very strongly disagree
1	⬭
2	⬭
3	⬭
4	⬭
5	⬭
6	⬭
7	⬭
	Very strongly agree

161. 161. The introduction of AI-based diagnostic modalities will require regulatory supervision, both related to the quality of the rendered diagnosis and the ultimate destination of the diagnostic information *

Mark only one oval.

Very strongly disagree	
1	◯
2	◯
3	◯
4	◯
5	◯
6	◯
7	◯

Very strongly agree

162. 162. Regulatory approval of AI tools used for definitive (primary) diagnosis will be very strict, but AI used for advisory purposes (secondary) will also have to meet strict regulatory conditions *

Mark only one oval.

	Very strongly disagree
1	⬭
2	⬭
3	⬭
4	⬭
5	⬭
6	⬭
7	⬭
	Very strongly agree

163. 163. CLIA regulations and clarification surrounding the use of laboratory data within pathology and laboratory processes versus outside of the laboratory will be reviewed and updated *

Mark only one oval.

	Very strongly disagree
1	◯
2	◯
3	◯
4	◯
5	◯
6	◯
7	◯
	Very strongly agree

164. 164. Governments will actively promote innovation in the areas of AI and medicine, fostering the advancement of AI in pathology *

Mark only one oval.

Very strongly disagree
1 ◯
2 ◯
3 ◯
4 ◯
5 ◯
6 ◯
7 ◯
Very strongly agree

165. 165. Legal disputes will often arise regarding who should assume liability (pathologist, institution, developer, commercial vendor...) for diagnostic errors induced by AI *

Mark only one oval.

Very strongly disagree

1 ⬭
2 ⬭
3 ⬭
4 ⬭
5 ⬭
6 ⬭
7 ⬭

Very strongly agree

Section 9: Ethical aspects

Please rate your AGREEMENT with the following statements according to this Likert scale:

1 Very strongly disagree
2 Strongly disagree
3 Disagree
4 Neither agree nor disagree
5 Agree
6 Strongly agree
7 Very strongly agree

By 2030, regarding the integration of AI in pathology ...

166. 166. As long as AI is used as a supportive method, ethical issues will be minor. However, when AI takes over tasks from the pathologist, i.e., making a diagnosis without human oversight, it will face major ethical challenges. *

Mark only one oval.

	Very strongly disagree
1	◯
2	◯
3	◯
4	◯
5	◯
6	◯
7	◯
	Very strongly agree

167. 167. Other healthcare professionals will start using AI tools to diagnose cases without the aid of a pathologist *

Mark only one oval.

Very strongly disagree

1 ⬭

2 ⬭

3 ⬭

4 ⬭

5 ⬭

6 ⬭

7 ⬭

Very strongly agree

168. 168. Pathologists will occasionally make diagnoses against their own judgment because of AI software recommendations *

Mark only one oval.

	Very strongly disagree
1	◯
2	◯
3	◯
4	◯
5	◯
6	◯
7	◯
	Very strongly agree

169. 169. Due to the "black box" nature of many AI tools, pathologists will often make diagnoses without enough clinical explainability *

Mark only one oval.

	Very strongly disagree
1	⬭
2	⬭
3	⬭
4	⬭
5	⬭
6	⬭
7	⬭
	Very strongly agree

170. 170. Due to ethical concerns, algorithms will be prevented from making decisions in the pathology setting without additional manual review by a pathologist *

Mark only one oval.

	Very strongly disagree
1	◯
2	◯
3	◯
4	◯
5	◯
6	◯
7	◯
	Very strongly agree

171. 171. Hurried pathologists will often take "shortcuts" by accepting AI interpretations without sufficient verification *

Mark only one oval.

Very strongly disagree

1 ⬭

2 ⬭

3 ⬭

4 ⬭

5 ⬭

6 ⬭

7 ⬭

Very strongly agree

172. 172. It will be impossible to ensure that pathologists take full responsibility for double-checking and confirming AI-rendered diagnoses *

Mark only one oval.

Very strongly disagree

1 ◯
2 ◯
3 ◯
4 ◯
5 ◯
6 ◯
7 ◯

Very strongly agree

173. 173. Pathologists will still be legally responsible for diagnoses made with the help of AI *

Mark only one oval.

Very strongly disagree
1 ⬭
2 ⬭
3 ⬭
4 ⬭
5 ⬭
6 ⬭
7 ⬭
Very strongly agree

174. 174. Potentially-biased algorithms due to lack of demographic diversity in training datasets will lead to diagnostic errors *

Mark only one oval.

	Very strongly disagree
1	⬭
2	⬭
3	⬭
4	⬭
5	⬭
6	⬭
7	⬭
	Very strongly agree

175. 175. Limits will be imposed to unintended discoveries enabled by AI *

Mark only one oval.

Very strongly disagree	
1	◯
2	◯
3	◯
4	◯
5	◯
6	◯
7	◯
Very strongly agree	

176. 176. Stricter limits will be imposed on commercialization of data *

Mark only one oval.

	Very strongly disagree
1	⬭
2	⬭
3	⬭
4	⬭
5	⬭
6	⬭
7	⬭
	Very strongly agree

177. 177. Lack of informed patient consent when using their data will be a common practice *

Mark only one oval.

Very strongly disagree

1 ⬭

2 ⬭

3 ⬭

4 ⬭

5 ⬭

6 ⬭

7 ⬭

Very strongly agree

178. 178. Data inferences that may impact on patient anonymity will lead to ethical issues *

Mark only one oval.

	Very strongly disagree
1	⬭
2	⬭
3	⬭
4	⬭
5	⬭
6	⬭
7	⬭
	Very strongly agree

179. 179. AI will lead to a de-skilling of pathologists, who will potentially suffer from too great a reliance on AI *

Mark only one oval.

	Very strongly disagree
1	⬭
2	⬭
3	⬭
4	⬭
5	⬭
6	⬭
7	⬭
	Very strongly agree

180. 180. AI and technology will be included in the educational curricula of medical students, pathologists, and analysts to help them deal with this rapidly evolving method of support and its ethical implications *

Mark only one oval.

	Very strongly disagree
1	◯
2	◯
3	◯
4	◯
5	◯
6	◯
7	◯
	Very strongly agree

Thank you for your participation!

You have finished Round 2 of the survey. We will process everyone's responses and will send you a link to the Round 3 survey by January 20, 2021.

Statements which do not reach consensus in Round 2 will be rated again. Thus, you will be shown median results from Round 2 and will be offered the option to change your ratings to the same statements, if you wish.

This content is neither created nor endorsed by Google.

Google Forms
